# IGF2BP3/ESM1/KLF10/BECN1 positive feedback loop: a novel therapeutic target in ovarian cancer via lipid metabolism reprogramming

**DOI:** 10.1038/s41419-025-07571-7

**Published:** 2025-04-17

**Authors:** Anbo Gao, Juan Zou, Tian Zeng, Mei Qin, Xing Tang, Ting Yi, Guangming Song, Jie Zhong, Yuhuan Zeng, Wenchao Zhou, Qin Gao, Qunfeng Zhang, Juan Zhang, Yukun Li

**Affiliations:** 1 Clinical Research Institute, The Second Affiliated Hospital, University of South China, Hengyang, Hunan China; 2 Hengyang Medical School, University of South China, Hengyang, Hunan China; 3 Department of Assisted Reproductive Centre, Zhuzhou Hospital Affiliated to Xiangya School of Medicine, Central South University, Zhuzhou, Hunan China; 4 Hunan Province Key Laboratory of Tumor Cellular & Molecular Pathology, Cancer Research Institute, Hengyang Medical School, University of South China, Hengyang, Hunan China; 5 Department of Gynecology, Zhuzhou Hospital Affiliated to Xiangya School of Medicine, Central South University, Zhuzhou, Hunan China; 6 Department of Gynecology, The Second Affiliated Hospital, University of South China, Hengyang, Hunan China; 7 Department of Trauma Center, Zhuzhou Hospital Affiliated to Xiangya School of Medicine, Central South University, Zhuzhou, Hunan China

**Keywords:** Ovarian cancer, Predictive markers, Ovarian cancer

## Abstract

Ovarian cancer (OC) is often detected at an advanced stage and has a high recurrence rate after surgery or chemotherapy. Thus, it is essential to develop new strategies for OC treatment. This study tended to investigate the effects of endothelial cell-specific molecule 1 (ESM1) in OC. The impact of ESM1 on lipid metabolism was investigated through the regulation of ESM1 expression. Differential genes regulated by ESM1 were screened by mRNA sequencing. The role of autophagy in ESM1 regulation on lipid metabolism was explored using autophagy inhibitor chloroquine (CQ). Co-IP, dual-luciferase reporter assay, actinomycin D treatment assay, and others were used to analyze the mechanism of ESM1 regulation on lipid metabolism. The xenograft mouse model was constructed to explore the impact of ESM1 regulation on OC development. The regulatory mechanism of ESM1 in OC patient samples was verified by using microarray analysis and the Log-rank (Mantel-Cox) test. After ESM1 silencing, cholesterol synthesis decreased and lipolysis increased. mRNA sequencing revealed that ESM1 regulation on lipid metabolism was related to Beclin 1 (BECN1). In vitro experiments, ESM1 inhibited lipolysis by suppressing BECN1-mediated autophagy. BECN1 expression was regulated by the transcription factor Kruppel-like factor 10 (KLF10). The competitive binding between BECN1 and HSPA5 promoted the ubiquitination degradation of HMGCR, thereby inhibiting cholesterol production. The intervention experiment with exogenous cholesterol showed a positive correlation between m6A reader IGF2BP3 expression and cholesterol content. Mechanistically, IGF2BP3 regulated the stability of ESM1 mRNA. In vivo experiments, ESM1 modified by m6A methylation promoted cholesterol synthesis and inhibited lipolysis. High expression of ESM1 predicted poor prognosis in OC patients. ESM1 regulated lipid metabolism through IGF2BP3/ESM1/KLF10/BECN1 positive feedback, which was a promising target for OC treatment.

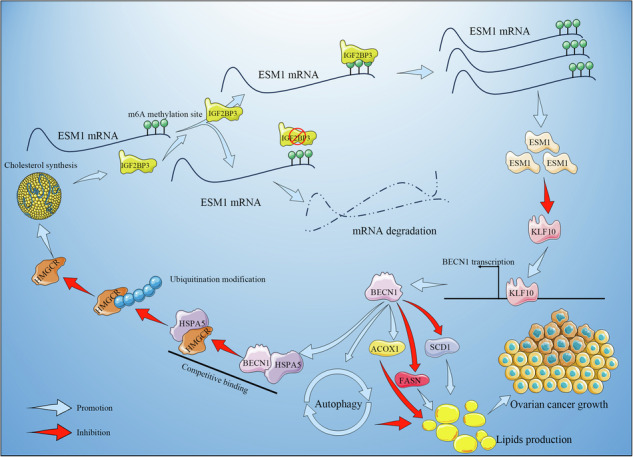

## Introduction

Ovarian cancer (OC) is a deadly gynecological cancer and a common cause of death among women worldwide [1]. Due to its asymptomatic nature and lack of effective early biomarkers, OC patients are often diagnosed in the late stages [2]. Although multiple drugs and treatment strategies have been developed to treat OC, such as surgery and chemotherapy, the recurrence rate of patients remains high [3]. Therefore, to develop promising diagnostic and therapeutic interventions, it is urgent to study the specific mechanisms underlying OC progression.

Endothelial cell-specific molecule 1 (ESM1), a 50 kDa secreted proteoglycan, plays a crucial role in regulating cell adhesion, inflammatory diseases, and tumor progression [4]. ESM1 is considered an oncogene in various cancers and a promising therapeutic target and prognostic indicator [5]. ESM1 is significantly overexpressed in colorectal cancer and has excellent predictive performance for patient survival [6]. Similarly, ESM1 is overexpressed in triple-negative breast cancer cell lines and patient tissues, which is linked to poor prognosis [7]. ESM1 is significantly upregulated in OC tissues and is also related to poor patient prognosis [8, 9]. Research has shown that ESM1 is closely related to the occurrence and development of OC, and can be regarded as a new biomarker and therapeutic target for OC patients [10, 11]. However, the specific carcinogenic mechanism of ESM1 in OC needs further clarification.

Lipid metabolism disorder has been recognized as a hallmark of human cancer [12, 13]. Cancer cells utilize lipid metabolism to obtain substances required for proliferation, metastasis, and their impact on the tumor microenvironment and response to cancer treatment [14]. Single-cell RNA sequencing was performed on different early stages of lung cancer, and it was found that lipid metabolism is widely disrupted in different types of cells [15]. Research has found that abnormal lipid metabolism is involved in the occurrence and progression of liver cancer, colorectal cancer, and breast cancer [16–19]. Therefore, targeted lipid metabolism is a promising strategy for treating human cancer [20]. In OC, lipid metabolism disorder promotes tumor development and drug resistance, which brings adverse prognoses and outcomes to patients [21]. Therefore, targeted regulation is essential for the survival and treatment of OC patients. In our previous research, the interaction between ANGPTL4 and ESM1 drove lipid metabolism reprogramming, promoting OC cell proliferation and invasion by inactivating lipoprotein lipase (LPL) [22]. Research has shown that autophagy defects are directly related to metabolic disorders. Autophagy regulates lipid metabolism through selective flipping of NCoR1 [23]. However, the specific mechanism by which ESM1 regulates lipid metabolism in OC still needs further clarification.

N6 methyladenosine (m6A), the most abundant RNA modification in mammals, plays an important role in many diseases, especially tumors [24]. More and more evidence suggests that changes in m6A levels are related to the occurrence, progression, or prognosis of OC [25]. IGF2BP3 can act as an m6A reader, stabilizing the mRNAs of many tumor-related genes [26]. Research has found that inhibition of OC progression can be achieved by disrupting the stability of IGF2BP3-targeted mRNA [27]. Knockdown of IGF2BP3 inhibits the growth of OC cells by regulating the stability of PLAGL2 mRNA [26]. However, there have been no reports on whether IGF2BP3 targets ESM1 mRNA stability in an m6A-dependent manner to improve lipid metabolism disorders in OC.

Based on the above research, this study focused on the IGF2BP3/ESM1/KLF10/BECN1 axis to explore the role and potential mechanisms of ESM1 in OC cells and animal models and was further verified in patient samples, providing new insights for the development of treatment methods for OC.

## Materials and methods

### Cell culture

A2780 cells (AW-CCH152, Abiowell) were cultured in RPMI-1640 medium (R8758, Sigma), and SKOV3 cells (AW-CCH110, Abiowell) were cultured in McCoy’s 5 A medium (ICell-0011, iCell). All mediums were supplemented with 10% fetal bovine serum (FBS, 10099141, Gibco) and 1% penicillin/streptomycin (SV30010, Beyotime). All cells were placed in a humidified incubator (DH-160I, SANTN) at 37 °C with 5% (v/v) CO_2_. All cells were identified by short tandem repeats (STR) and verified free of mycoplasma contamination.

### Overexpression and silencing of target genes

According to the manufacturer’s instruction, sh-ESM1, oe-ESM1, sh-BECN1, oe-BECN1, sh-HSPA5, oe-HSPA5, sh-IGF2BP3, oe-IGF2BP3, and their negative controls (HonorGene) were transfected in OC cells using Lipofectamine 2000 (11668019, Invitrogen). After transfection for 24 h, OC cells were collected for subsequent analysis.

### Cell treatment

To exclude the influence of secretory ESM1 on lipid metabolism, OC cells were treated with normal goat IgG or anti-ESM1 neutralizing antibody (0.2 mg/mL) for 24 h [28]. To verify the necessity of autophagy in ESM1 regulation of lipid metabolism in OC cells, OC cells were treated with 25 µM chloroquine (CQ) for 48 h [29]. To investigate whether BECN1/HSPA5 regulates the ubiquitination of HMGCR, 5 μM proteasome inhibitor MG132 was utilized to treat OC cells for 2 h [30]. To explore the effects of cholesterol content on mRNA stability and protein expression of ESM1 and IGF2BP3, after incubation with or without 20 µM Pravastatin (Pra) for 24 h, various concentrations of soluble cholesterol-MβCD complexes (mβCD-chol) (0, 0.5, 1, 2.5, 5, 10, and 20 μg/mL) were used to intervene OC cells for 1 h [31]. Actinomycin D treatment assay was applied to analyze the stability of ESM1 mRNA in OC cells after the above treatment. The procedure was described in the literature [32].

### Construction of xenograft mouse model

Female BALB/c nude mice (4 weeks) were ordered from Hunan SJA Laboratory Animal Co., Ltd. Each group contained five mice. The sample size was calculated using efficacy analyses [33]. After one week of adaptive feeding, 1 × 10^6^ A2780 cells transfected with/without sh-ESM1, oe-ESM1, sh-IGF2BP3, and oe-IGF2BP3 were injected subcutaneously into the armpit of the foreright limb of nude mice [10]. The body weight of mice was measured twice a week. The experiment was stopped when the maximum tumor volume reached 1 cm^3^ (about four weeks). The peripheral blood was taken from the nude mice after anesthesia. Finally, 150 mg/kg pentobarbital sodium was injected intraperitoneally for euthanasia [34]. All procedures were approved by the research ethics committee of Zhuzhou Hospital Affiliated to Xiangya School of Medicine with Institutional Review Board (IRB) approval (#ZZCHEC2023029-01).

### Quantitative real-time polymerase chain reaction (RT-qPCR)

Samples were treated with Trizol reagent (15596026, Thermo Fisher Scientific) to obtain total RNA. Then, RNA was reversely transcribed into cDNA using an mRNA reverse transcription kit (CW2569, CWBIO). RT-qPCR was carried out using UltraSYBR Mixture (CW2601, CWBIO). The relative expressions of genes were calculated by the 2^−△△Ct^ method. The endogenous control gene was β-actin. The primers are listed in Table 1.

**Table 1:** Sequences of the primers.

Gene name	Forward (5’-3’)	Reverse (5’-3’)
H-β-actin	ACCCTGAAGTACCCCATCGAG	AGCACAGCCTGGATAGCAAC
H-ESM1	TTGCTACCGCACAGTCTCAG	GCAGGTCTCTCTGCAATCCA
H-BECN1	CATGGAGAACCTCAGCCGAA	ACAGCGTTTGTAGTTCTGACAC
H-KLF10	CTTCCGGGAACACCTGATTTT	GCAATGTGAGGTTTGGCAGTATC

### Western blot

Total proteins were extracted from samples using radioimmunoprecipitation assay (RIPA) lysis buffer (AWB0136, Abiowell). BCA protein quantification kit (AWB0104, Abiowell) was utilized to analyze the protein concentration. The proteins were isolated by 10% SDS-PAGE and transferred onto nitrocellulose (NC) membranes. After being blocked in 5% skimmed milk (AWB0004, Abiowell) for 1.5 h, membranes were immersed in solutions of primary antibodies at 4 °C overnight, followed by the secondary antibodies for 1.5 h. All the membranes were added with Super ECL Plus detection reagent (AWB0005, Abiowell) for chemiluminescence imaging (ChemiScope6100). Quantity One 4.6.6 (Bio-Rad Inc.) was utilized to analyze the gray values of protein bands. The internal reference was β-actin. The information on antibodies is exhibited in Table 2. Full and uncropped western blots are displayed in Supplementary Material.

**Table 2:** The information on antibodies.

Names	Item number	dilution rate	Companies
ESM1	ab103590	1: 1000	UK, Abcam
FASN	10624-2-AP	1: 20000	China, Proteintech
SCD1	ab19862	1: 1000	UK, Abcam
HMGCS1	ab155787	1: 1000	UK, Abcam
HMGCR	ab242315	1 µg/mL	UK, Abcam
CD36	18836-1-AP	1: 1000	China, Proteintech
ACC1	21923-1-AP	1: 5000	China, Proteintech
CPT1A	ab234111	1: 1000	UK, Abcam
CPT2	ab181114	1: 5000	UK, Abcam
ACOX1	68017-1-Ig	1: 5000	China, Proteintech
IGF2BP3	14642-1-AP	1: 5000	China, Proteintech
SREBP1	ab28481	1: 2000	UK, Abcam
SREBP2	ab30682	1: 1000	UK, Abcam
β-actin	66009-1-Ig	1: 5000	China, Proteintech
BECN1	11306-1-AP	1: 6000	China, Proteintech
HSPA5	66574-1-Ig	1: 20000	China, Proteintech
HMGCR	AWA10042	1: 1000	China, Abiowell
PCNA	10205-2-AP	1: 5000	China, Proteintech
HRP goat anti-mouse IgG	SA00001-1	1: 5000	China, Proteintech
HRP goat anti-rabbit IgG	SA00001-2	1: 6000	China, Proteintech

### Cell counting kit-8 (CCK-8) assay

Cells were digested with trypsin (AWC0238, Abiowell) and cultured at 37 °C (2 × 10^4^ cells/well). After adhesion, they were treated accordingly for 48 h. Next, 10 μL of CCK-8 reagent (NU679, DOJINDO) replaced the original medium. Finally, OC cells were cultured for 4 h, and then optical density (OD) at 450 nm was read by a multifunctional microplate reader (MB-530, HEALES).

### Quantification of neutral lipid

Intracellular neutral lipid content was measured using the cell-permeable fluorescent lipophilic dye BODIPY 493/503(D3922, Thermo Fisher Scientific) [35]. 2 × 10^5^ cells were fixed in 4% paraformaldehyde for 15 min and incubated with 1 μg/mL BODIPY 493/503 at 37 °C for 1 h. After using Hoechst nucleus redyeing, Image J software (http://imagej.nih.gov/ij/) was employed to quantify the lipid staining intensity of images.

### Determination of triglyceride (TG), total cholesterol (TC), and non-esterified fatty acids (NEFA)

The different treated cells were lyzed in RIPA buffer for 40 min. Then, cell homogenates were prepared using chloroform/methanol (2:1) for lipid extraction [35]. According to the manufacturer’s instruction, TG (A110-1-1, NJJCBIO), TC (A111-1-1, NJJCBIO), and NEFA (A042-2-1, NJJCBIO) kits were used to determine the levels of TG, TC, and NEFA, respectively.

### mRNA sequencing

According to the requirements of the Beijing Genomics institution (BGI), SKOV3 cell samples transfected with sh-NC and sh-ESM1 were broken and split, purified and RNA collected. The samples were then subjected to sample quality control, mRNA isolation, mRNA fragmentation, cDNA synthesis, end repair, adaptor ligation, PCR, library quality control, cyclization, and sequencing. For more information, please refer to the guidelines of BGI (https://www.yuque.com/yangyulan-ayaeq/oupzan/lmx609).

### Fluorescence in situ hybridization (FISH) and immunofluorescence (IF) analysis

The distribution of ESM1 mRNA in OC cells was detected by the FISH probe. The cell slides were fixed with 4% paraformaldehyde for 30 min, treated with 0.3% Triton X-100, and permeated at 37 °C for 30 min. At the same time, the hybrid solution was preheated at 37 °C. Under the condition of avoiding light, 2.5 μL of 100 μM lncRNA FISH Probe Mix storage solution or internal reference FISH Probe Mix storage solution was added to 100 μL of hybrid solution. After discarding the pre-hybridization solution in each well, 100 μL of probe hybridization solution was added and hybridized at 37 °C overnight. The cell slides were rinsed with 4×, 2×, and 1× saline-sodium citrate buffer successively to reduce the background signal. The expression of IGF2BP3 in OC cells was detected by IF staining. The cell slides were fixed with 4% paraformaldehyde for 20 min and rinsed with PBS for 5 min. IGF2BP3 monoantibody was added to cell slides and incubated at 4 °C overnight. 50 ~ 100 μL of anti-rabbit IgG labeled fluorescent antibody was added, incubated at 37 °C for 90 min, and washed with PBS for 5 min. The cell slides were sealed with 90% glycerin and observed under a fluorescence microscope (BA410E, Motic). ESM1 mRNA positive staining is red fluorescence, IGF2BP3 positive staining is green fluorescence, and blue staining is DAPI nucleation.

### Dual-luciferase reporter assay

The activity of BECN1 promoter and the target of KLF10 and BECN1 promoter were analyzed by dual-luciferase reporter assay. HEK-293T cells (AW-CNH086, Abiowell) were co-transfected with psiCHECK-2-BECN1-wt, psiCHECK-2-BECN1-Mut1 (Mutation site - CCCCCCCCCCCC), psiCHECK-2-BECN1-Mut2 (Mutation site - TATCCGCACGGGGTGGTGCGGGC) (HonorGene) along with sh-NC, sh-ESM1, oe-NC, or oe-KLF10. After being cultured for 48 h, cells were lysed and treated by using a dual-luciferase reporter assay kit (E1910, Promega). The activity of firefly luciferase and Renilla luciferase was measured using a chemiluminescence detector (GloMax 20/20, Promega).

### Co-immunoprecipitation (Co-IP)

Co-IP was applied to detect the interaction relationship among BECN1, HSPA5, and HMGCR in OC cells. 20 μL of Protein A/G agarose beads were mixed with 200 μL of IP lysate, followed by centrifugation at 3000 rpm for 3 min to retain precipitation. The cell lysate incubated with the antibody overnight was added to the pre-treated Protein A/G agarose beads and slowly shaken at 4 °C for 2 h to fully conjugate the antibody and Protein A/G agarose beads. Then, agarose beads precipitate was collected by centrifugation at 3000 rpm for 3 min. BECN1 or HSPA5 antibody was utilized as the decoy protein, and the protein expressions of BECN1, HSPA5, and HMGCR in OC cells were detected by western blot.

### Multiplex IF analysis of OC tissue microarray

The OC tissue chip (AF-OvaSur2201) was purchased from AiFang Biological and included 94 cancer tissues. The clinical characteristics of the patient, including age, pathological grade, TNM staging data, and treatment methods, are presented in Table 3. The levels of ESM1, IGF2BP3, KLF10, BECN1, HMGCR, and SCD1 were measured utilizing multiplex IF as described in the literature [36].

**Table 3:** The relationship between the expression of ESM1 and the clinicopathological features of OC patients.

Items	ESM1-L	ESM1-H	*p-*value
***N***	31	63	
**Age (mean** **±** **SD)**	53.46 ± 10.85	53.33 ± 8.64	0.951
**T stage**			0.3537
T1	3	8	
T2	5	4	
T3	23	49	
T4	0	2	
**N stage**			0.5893
N0	20	37	
N1	11	26	
**M stage**			0.319
M0	28	52	
M1	3	11	
**FIGO stage**			0.7381
I	3	8	
II	4	4	
III	18	39	
IV	6	12	
**Methods of treatment**			0.0869
Surgery	12	13	
Chemotherapy	11	34	
Surgery plus chemotherapy	4	13	
Other	4	3	

The grouping of ESM1 high and low expression was based on the best cut-off point using X-tile. FIGO, Federation International of Gynecology and Obstetrics.

### RNA m6A modification quantification

RNA Immunoprecipitation Kit (Sigma) was applied to evaluate the m6A levels of ESM1 mRNA. Briefly, the magnetic beads were mixed with 5 μg of m6A monoclonal antibody (68055-1-Ig, Proteintech) and IgG antibody prior to the addition of cell lysates (2 × 10^7^ cells per sample). After proteinase K treatment, RNA was eluted from the immunoprecipitation complexes and purified for further analysis of ESM1 mRNA levels using qPCR.

### Data analysis

Data were analyzed using GraphPad Prism 8.0 (GraphPad Software Inc.) and presented as mean ± standard deviation (SD). Comparisons between two groups were performed using a two-tailed unpaired Student’s *t*-test. One-way analysis of variance (ANOVA) and Tukey’s post hoc tests were applied for comparisons amongst multiple groups. Data comparisons between groups at different time points were analyzed by two-way ANOVA with Bonferroni as a post hoc test. The comparison of survival curves was conducted by the Log-rank (Mantel-Cox) test. All experiments were randomized and blindly analyzed to minimize experimental bias. Statistical significance was defined as *p* < 0.05.

## Results

### ESM1 silencing reduced cholesterol synthesis and promoted lipolysis in OC cells

**Fig. 1:**
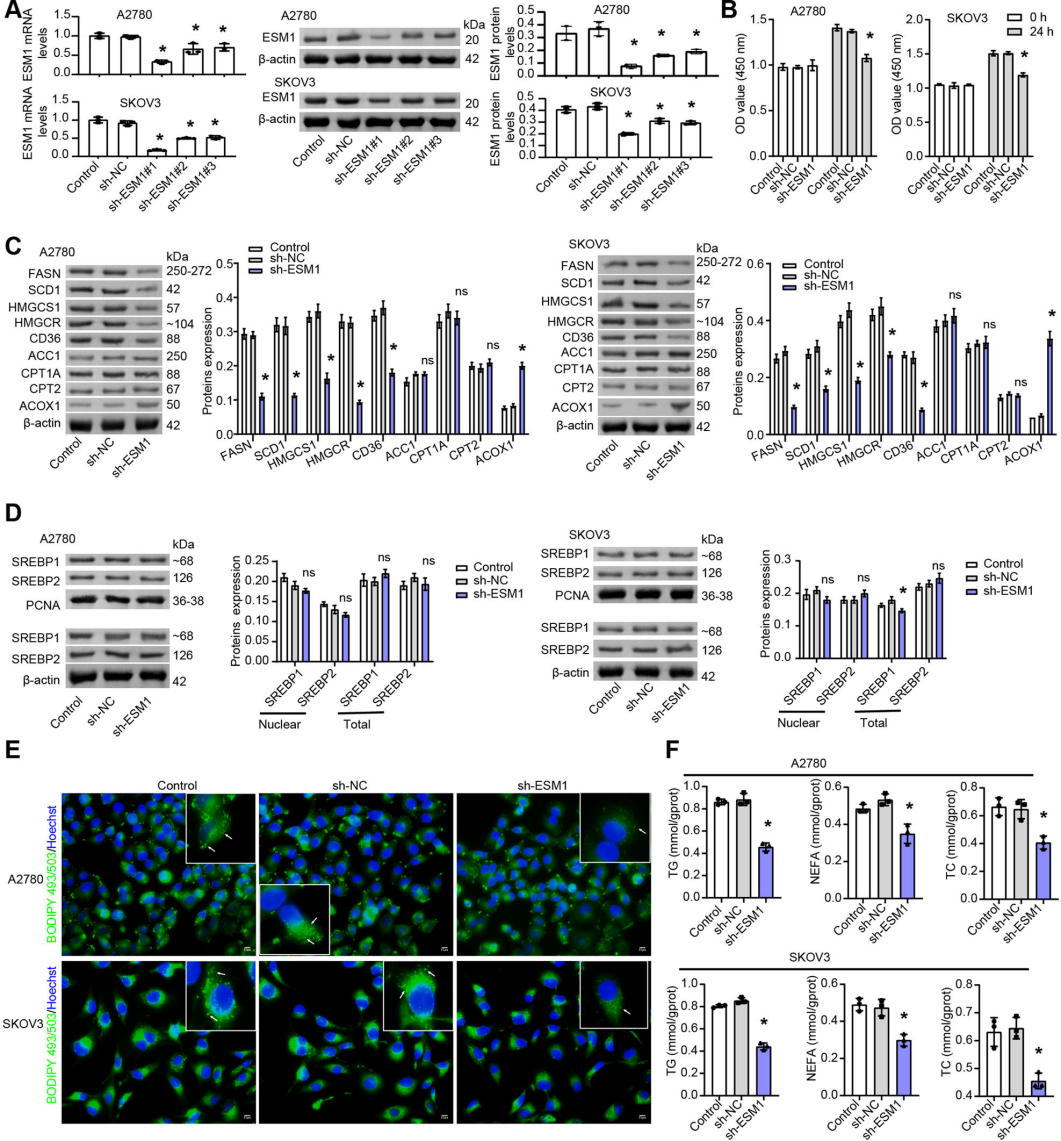
In OC cells, inhibition of ESM1 expression reduced cholesterol synthesis and promoted lipolysis. **A** The mRNA and protein levels of ESM1 in OC cells were analyzed by RT-qPCR and western blot. **B** The viability of OC cells was detected by CCK-8. **C** Western blot analysis of ACC1, FASN, SCD1, HMGCS1, HMGCR, CD36, CPT1A, CPT2, and ACOX1 levels in OC cells. **D** Western blot analysis of the nuclear and total levels of SREBP1 and SREBP2 in OC cells. **E** Neutral lipid content in OC cells was observed by BODIPY 493/503 staining. The white arrows point to the locations where neutral lipid droplets accumulate. Scale bar, 25 μm. **F** Biochemical kits were applied to assess the contents of TG, NEFA, and TC in OC cells. *p < 0.05 vs. sh-NC.

Studies have shown that dysregulation of lipid metabolism affects the development of OC [35, 37]. Our previous work found that ESM1 was related to lipid metabolism [22]. Therefore, we wanted to investigate the effect of ESM1 expression on lipid metabolism in OC cells. Since ESM1 was a secreted protein, we first blocked secreted ESM1 using neutralizing antibodies to analyze the types of ESM1 that affected lipid metabolism. The results in Fig. S1 showed that the addition of neutralizing antibodies had no significant effect on the level of lipid metabolism markers compared with IgG. This suggested that it was not secretory ESM1 that influenced lipid metabolism, but the accumulation of intracellular ESM1. Consequently, we next focused on the impact of intracellular ESM1 levels on lipid metabolism. In OC cells stably transfected with sh-ESM1 (#1-3), sh-ESM1#1 showed the best silencing efficiency. Thus, sh-ESM1#1 was selected for the subsequent ESM1 silencing experiment (Fig. 1A). ESM1 silencing reduced OC cell viability (Fig. 1B). Further, by western blot assay, we analyzed the effect of ESM1 silencing on fatty acid synthesis (ACC1, FASN, and SCD1), cholesterol biosynthesis (HMGCS1 and HMGCR), fatty acid uptake (CD36), fatty acid oxidation (CPT1A), and lipid catabolism (CPT2 and ACOX1)-related indicators. The results displayed that ESM1 silencing down-regulated the levels of FASN, SCD1, HMGCS1, HMGCR, and CD36 in OC cells, and up-regulated the levels of ACOX1. However, ESM1 silencing had no significant effect on levels of ACC1, CPT1A, and CPT2 (Fig. 1C). SREBP1 and SREBP2 are major transcription factors controlling fatty acid and cholesterol biosynthetase, and it has been confirmed that their up-regulation promotes tumor progression [38, 39]. Western blot results illustrated that ESM1 silencing had no significant effect on nuclear and total levels of SREBP1 and SREBP2, suggesting that ESM1 did not drive lipid synthesis in tumor progression by promoting nuclear accumulation of SREBPs (Fig. 1D). Intracellular neutral lipid staining with BODIPY 493/503 in OC cells showed that ESM1 silencing significantly reduced neutral lipid content in OC cells (Fig. 1E). Moreover, the contents of TG, NEFA, and TC in OC cells were all decreased after ESM1 silencing (Fig. 1F). The above suggested that ESM1 played a leading role in controlling lipid metabolism and that ESM1 silencing could reduce cholesterol synthesis and promote lipolysis in OC cells.

### ESM1 regulation of lipid metabolism in OC was related to BECN1

**Fig. 2:**
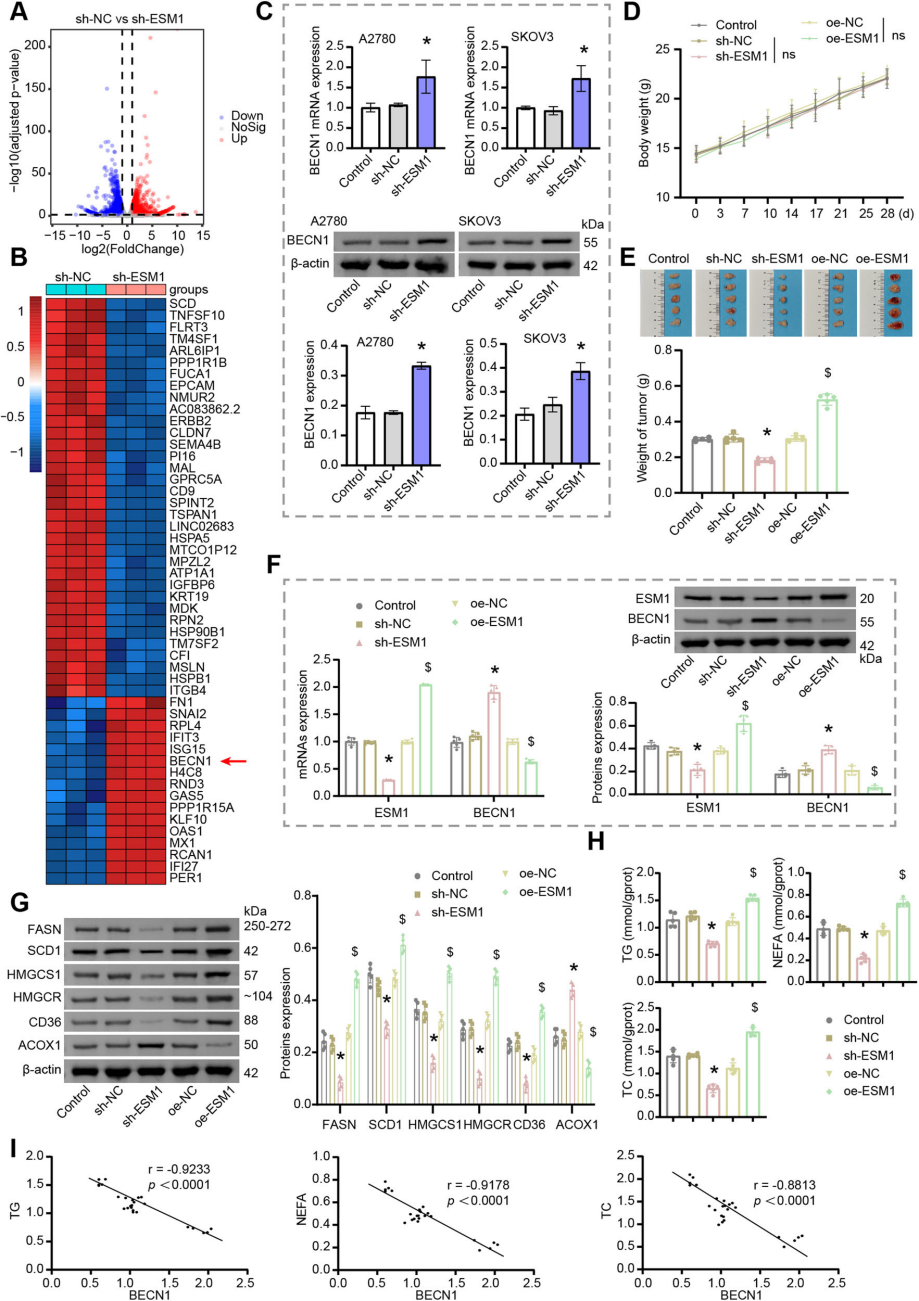
The regulation of lipid metabolism in OC by ESM1 was related to BECN1. **A** Visualization of DEGs in SKOV3 cells with volcano plot. **B** Heat map visualization of DEG expression in SKOV3 cells. **C** The mRNA and protein levels of BECN1 in OC cells were analyzed by RT-qPCR and western blot. **D** Growth curve of nude mice. **E** The weight of the tumor. **F** The mRNA and protein levels of ESM1 and BECN1 in tumor tissues were detected by RT-qPCR and western blot. **G** Western blot analysis of FASN, SCD1, HMGCS1, HMGCR, CD36, and ACOX1 levels in tumor tissues. **H** Biochemical kits were utilized to measure the contents of TG, NEFA, and TC in the serum. **I** The correlation between BECN1 level and TG, NEFA, and TC levels in the serum was analyzed by the Pearson correlation coefficient. *p < 0.05 vs. sh-NC. ^$^*p* < 0.05 vs. oe-NC.

To further explore the internal pathway of ESM1 regulation of lipid metabolism in OC cells, mRNA sequencing was further performed after ESM1 silencing of SKOV3 cells. The volcano map showed differentially expressed genes (DEGs) regulated by ESM1 (Fig. 2A). The heat map further visualized DEG expression in ESM1-silenced SKOV3 cells. The results displayed that BECN1 expression, which was associated with autophagy, was elevated after ESM1 silencing (Fig. 2B). Results of RT-qPCR and western blot further verified the upregulation of BECN1 expression in OC cells by ESM1 silencing (Fig. 2C). A study has shown that autophagy is involved in regulating lipid metabolism [23]. Furthermore, functional enrichment analysis revealed that ESM1 is associated with the cellular process of the lysosome, which is involved in the degradation of proteins mediated by the autophagy-lysosome system (Fig. S2). Therefore, BECN1 was selected for subsequent analysis. Subsequently, we conducted animal experiments to further verify the in vivo impacts of ESM1 silencing on OC progression and the impacts of BECN1 on ESM1 regulation of lipid metabolism. Within 28 days after injection of A2780 cells treated with or without sh-ESM1 and oe-ESM1, the body weight of nude mice gradually increased over time without significant differences between groups (Fig. 2D). Additionally, ESM1 silencing reduced tumor weight, while ESM1 overexpression did the opposite, suggesting that ESM1 could promote tumorigenicity in A2780 cells in vivo (Fig. 2E). Consistent with the results of cell experiments, ESM1 silencing in vivo also upregulated BECN1 expression in tumors at the transcriptional and translational levels, while ESM1 overexpression showed the opposite trend (Fig. 2F). Further, by western blot analysis of lipid metabolism-related indicators, we found that ESM1 silencing down-regulated FASN, SCD1, HMGCS1, HMGCR, and CD36 expressions in tumor tissues, and up-regulated the expression of ACOX1. However, the expression of these proteins displayed an opposite trend when ESM1 was overexpressed (Fig. 2G). Moreover, the contents of TG, NEFA, and TC in the serum of nude mice decreased after ESM1 silencing but increased after ESM1 overexpression (Fig. 2H). Pearson correlation coefficient revealed a significant negative correlation between BECN1 expression and TG (*r* = −0.9233, *p* < 0.0001), NEFA (*r* = −0.9178, *p* < 0.0001), and TC (*r* = −0.8813, *p* < 0.0001) contents (Fig. 2I). In conclusion, these results suggested that ESM1 could regulate lipid metabolism in vivo to promote OC progression, and this regulation might be related to the abnormal expression of BECN1.

### BECN1 was involved in ESM1 regulation of lipolysis and cholesterol production in OC cells

**Fig. 3:**
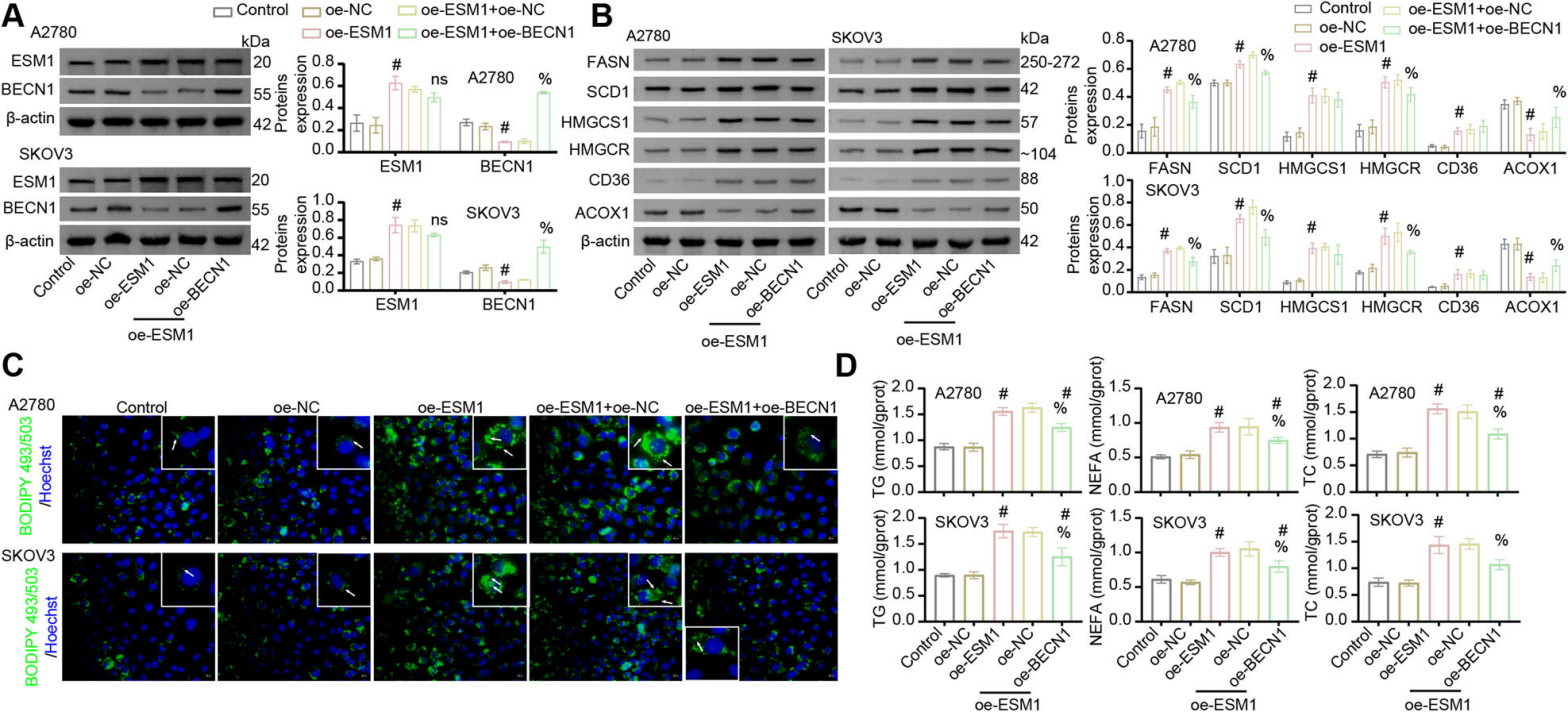
BECN1 was involved in ESM1 regulation of lipid metabolism in OC cells. **A** Western blot was utilized to verify the overexpression efficiency of ESM1 and BECN1. **B** Western blot analysis of FASN, SCD1, HMGCS1, HMGCR, CD36, and ACOX1 levels in OC cells. **C** The content of neutral lipids in OC cells was observed by BODIPY 493/503 staining. The white arrows point to the locations where neutral lipid droplets accumulate. Scale bar, 25 μm. **D** Biochemical kits were utilized to analyze the contents of TG, NEFA, and TC in OC cells. ^#^*p* < 0.05 vs. oe-NC. ^%^*p* < 0.05 vs. oe-ESM1+oe-NC.

To further explore the impact of BECN1 on ESM1 regulation of lipid metabolism in OC, we overexpressed ESM1 and BECN1 in OC cells. As shown in Fig. 3A, co-transfection of the ESM1 overexpression plasmid with the control plasmid was as effective as transfection of the ESM1 overexpression plasmid alone. In addition, transfection of the BECN1 overexpression plasmid did not affect the expression level of ESM1, however significantly rescued the intracellular level of BECN1 which was downregulated by ESM1 overexpression. Western blot analysis of relevant indicators of lipid metabolism showed that ESM1 overexpression promoted FASN, SCD1, HMGCS1, HMGCR, and CD36 expressions in OC cells, and inhibited the expression of ACOX1. However, BECN1 overexpression reversed the promotion of lipolysis and cholesterol production by ESM1 overexpression in OC cells (Fig. 3B). Furthermore, BODIPY 493/503 staining showed that ESM1 overexpression promoted the accumulation of neutral lipids in OC cells, but this situation was inhibited by BECN1 overexpression to some extent (Fig. 3C). Consistent with this, ESM1 overexpression increased the contents of TG, NEFA, and TC in OC cells. However, BECN1 overexpression partially inhibited the accumulation of TG, NEFA, and TC induced by ESM1 overexpression in OC cells (Fig. 3D). Compared with the oe-NC group, co-overexpression of ESM1 and BECN1 similarly promoted neutral lipid accumulation and increased intracellular contents of TG, NEFA, and TC. The above proved that BECN1 partially inhibits ESM1-mediated regulation of lipid metabolism in OC cells.

### ESM1 inhibited lipolysis in OC cells by suppressing BECN1-mediated autophagy

**Fig. 4:**
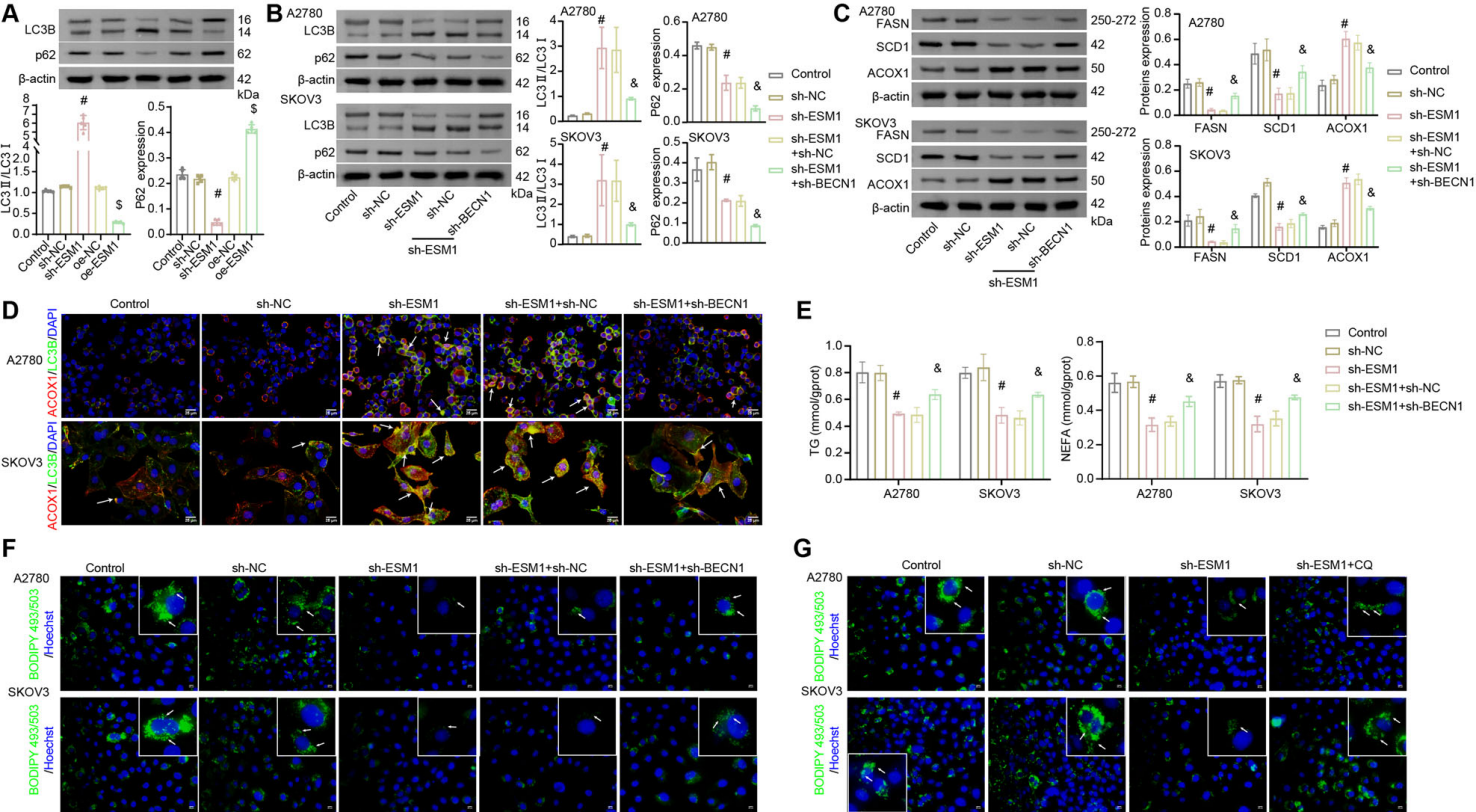
ESM1 inhibited lipolysis in OC cells by suppressing BECN1-mediated autophagy. **A**–**C** Western blot analysis of LC3B, p62, FASN, SCD1, and ACOX1 levels in OC cells. **D** The expressions of LC3B and ACOX1 in OC cells were analyzed by IF staining. White arrows represent the co-localization of LC3B and ACOX1. **E** Biochemical kits were utilized to assess the contents of TG and NEFA in OC cells. **F**, **G** The content of neutral lipids in OC cells was observed by BODIPY 493/503 staining. The white arrows point to the locations where neutral lipid droplets accumulate. ^#^*p* < 0.05 vs. sh-NC. ^$^*p* < 0.05 vs. oe-NC. ^&^*p* < 0.05 vs. sh-ESM1+sh-NC.

Previous studies have shown that activation of autophagy can inhibit lipid accumulation, and promote fatty acid oxidation and lipid droplet degradation [29, 40, 41]. Therefore, we conducted experiments to explore the role of ESM1 expression on BECN1-mediated autophagy. Western blot analysis of key markers of autophagy showed that ESM1 silencing significantly improved the autophagy level of OC cells, which was manifested as an increase in LC3 II/LC3 I level and a decrease in p62 level. However, ESM1 overexpression inhibited autophagy levels in OC cells (Fig. 4A). To further explore the role of BECN1-mediated autophagy in ESM1 regulation of lipid metabolism in OC cells, we silenced BECN1 in OC cells on the base of ESM1 silencing. BECN1 silencing reversed the increase in the autophagy level of OC cells induced by ESM1 silencing, which was manifested as a decrease in LC3 II/LC3 I levels (Fig. 4B). It was found that co-transfection of ESM1 silencing plasmid and control plasmid was as effective as transfection of ESM1 silencing plasmid alone. Further, ESM1 silencing down-regulated the expressions of FASN and SCD1 in OC cells, while ACOX1 showed the opposite trend. However, this change in lipid metabolism level was reversed by BECN1 silencing (Fig. 4C). Through IF staining, we found that the co-expression of ACOX1 and LC3B increased after ESM1 silencing and decreased after further BECN1 silencing (Fig. 4D). Additionally, the levels of TG and NEFA in OC cells decreased after ESM1 silencing and increased after further BECN1 silencing (Fig. 4E). Accordingly, the fluorescence intensity of BODIPY 493/503 decreased significantly after ESM1 silencing but rebounded after further BECN1 silencing (Fig. 4F). To verify the necessity of autophagy in ESM1 regulation of lipid metabolism in OC cells, we selected ESM1 silencing and autophagy inhibitor CQ for further analysis of OC cells. ESM1 silencing reduced the accumulation of neutral lipids in OC cells. However, the use of autophagy inhibitor CQ reversed the silencing effect of ESM1, and the accumulation of neutral lipids increased in OC cells (Fig. 4G). To summarize, these results suggested that ESM1 inhibited lipolysis in OC cells by suppressing BECN1-mediated autophagy.

### BECN1 regulated by KLF10 was involved in ESM1 regulation of lipid metabolism in OC cells

To further explore the internal mechanism of ESM1 regulating BECN1 expression, we analyzed the activity of the *BECN1* promoter in OC cells after silencing ESM1. Dual-luciferase reporter assay results displayed that ESM1 silencing enhanced the promoter activity of *BECN1* (Fig. S3A). Therefore, ESM1 might inhibit BECN1 expression by regulating the activity of a certain transcription factor. Combined with Fig. 2B, ESM1 silencing led to an upregulation of KLF10 gene expression, which exhibited a positive correlation with BECN1 levels. Furthermore, KLF10 was predicted to target BECN1 according to analyses conducted using the hTFtarget online software (http://bioinfo.life.hust.edu.cn/hTFtarget#!/). Thus, we speculated that ESM1 might inhibit BECN1 expression by inhibiting the activity of the transcription factor KLF10. RT-qPCR and western blot results together confirmed that ESM1 silencing promoted KLF10 transcription and translation (Fig. S3B). However, overexpression of ESM1 down-regulated KLF10 protein expression in tumor tissues of nude mice (Fig. S3C). Next, we transfected KLF10 overexpression plasmid into OC cells that had overexpressed ESM1 and performed BODIPY 493/503 staining observation and TC content assay. As shown in Fig. S3D-E, KLF10 overexpression partially reversed ESM1 overexpression-induced neutral lipid and cholesterol contents. To further verify the regulation of KLF10 on BECN1, we carried out a ChIP-qPCR assay to analyze multiple sites of the BECN1 promoter (#1-3). The results showed that there was an interaction between KLF10 and BECN1 promoter site 2 (Fig. S3F). The results of the dual-luciferase reporter assay further confirmed that KLF10 significantly upregulated BECN1 expression, indicating the positive targeted regulation of KLF10 on BECN1 expression (Fig. S3G). Subsequently, we further transfected BECN1 silencing plasmid into OC cells. It was revealed that BECN1 silencing completely reversed the neutral lipid and cholesterol accumulation inhibited by KLF10 overexpression (Fig. S3H-I). These results suggested that BECN1 regulated by KLF10 was involved in ESM1 regulation of lipid metabolism in OC cells.

### BECN1 combined with HSPA5/HMGCR to inhibit cholesterol production in OC cells

**Fig. 5:**
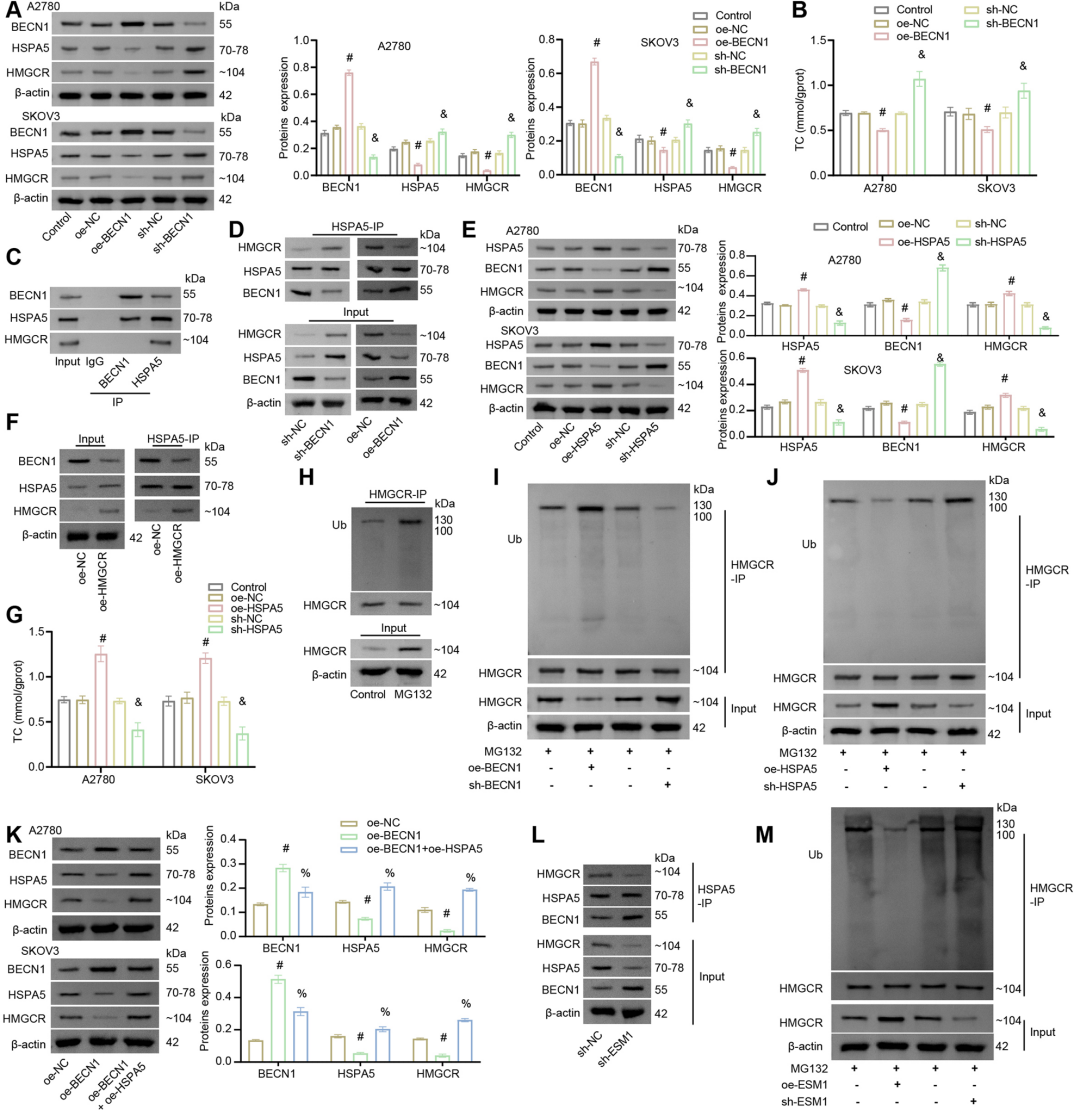
BECN1 combined with HSPA5/HMGCR to inhibit cholesterol production in OC cells. **A** Western blot analysis of BECN1, HSPA5, and HMGCR levels in OC cells. **B** A biochemical kit was utilized to assess the content of TC in OC cells. **C** Co-IP identification of the interaction between BECN1, HSPA5, and HMGCR. **D** Co-IP identification of the interaction among HSPA5, BECN1, and HMGCR. **E** Western blot detection of BECN1, HSPA5, and HMGCR levels in OC cells. **F** Co-IP identification of the interaction among HSPA5, BECN1, and HMGCR. **G** A biochemical kit was utilized to assess the content of TC in OC cells. ^#^*p* < 0.05 vs. oe-NC. ^&^*p* < 0.05 vs. sh-NC. **H**–**J** The ubiquitination modification level of HMGCR in SKOV3 cells was identified by IP. **K** Western blot detection of BECN1, HSPA5, and HMGCR levels in OC cells. **L** Co-IP identification of the interaction among HSPA5, BECN1, and HMGCR. ^#^*p* < 0.05 vs. oe-NC. ^%^*p* < 0.05 vs. oe-BECN1. **M** The ubiquitination modification level of HMGCR in SKOV3 cells was identified by IP.

Then, we analyzed the downstream pathway of BECN1 involved in lipid metabolism in OC cells. Based on the DEGs top50 in Fig. 2B and the differentially expressed proteins related to lipid metabolism (FASN, SCD1, HMGCS1, HMGCR, ACOX1, and CD36) in Fig. 2G, it was found that BECN1 might interact with HSPA5 and HSPA5 with HMGCR through the online prediction of STRING software. We verified the overexpression and silencing efficiency of BECN1 in OC cells by RT-qPCR (Fig. S4A). BECN1 overexpression down-regulated the expressions of HSPA5 and HMGCR in OC cells, while BECN1 silencing showed an opposite trend (Fig. 5A). In addition, BECN1 overexpression reduced TC accumulation in OC cells, while BECN1 silencing promoted TC accumulation in OC cells (Fig. 5B). Co-IP verified the interaction between BECN1 and HSPA5 as well as the interaction between HSPA5 and HMGCR (Fig. 5C). We subsequently discovered that the binding strength of HSPA5 to HMGCR was enhanced by BECN1 silencing, however, BECN1 overexpression inhibited the binding of HSPA5 to HMGCR (Fig. 5D). To further analyze whether the interaction between BECN1/HSPA5/HMGCR required the participation of HSPA5, we regulated the expression of HSPA5 in OC cells. We verified the overexpression and silencing efficiency of HSPA5 in OC cells by RT-qPCR (Fig. S4B). Western blot results displayed that HSPA5 overexpression down-regulated BECN1 expression and up-regulated HMGCR expression. However, the effect of HSPA5 silencing on these proteins showed an opposite trend (Fig. 5E). Moreover, HMGCR overexpression demonstrated an inhibitory effect on HSPA5 binding to BECN1 (Fig. 5F). Further assay by biochemical kits showed that HSPA5 silencing inhibited the accumulation of TC in OC cells (Fig. 5G). These findings suggest that BECN1 may regulate HMGCR expression by competitively binding to HSPA5.

Additionally, the intrinsic pathways by which BECN1/HSPA5 regulates HMGCR expression need to be further analyzed. Utilizing the qPTM online website (http://qptm.omicsbio.info) prediction was revealed that HMGCR undergoes ubiquitination modification. Next, we explore whether BECN1/HSPA5 regulates HMGCR expression through ubiquitination modification. In SKOV3 cells, HMGCR underwent ubiquitination modification (Fig. 5H). Both BECN1 overexpression and HSPA5 silencing were able to promote the ubiquitination of HMGCR, in contrast to BECN1 silencing and HSPA5 overexpression, which were able to inhibit its ubiquitination (Fig. 5I-J). Moreover, HSPA5 overexpression reversed the BECN1 overexpression-induced elevation of BECN1 expression and reduction of HSPA5 and HMGCR expression (Fig. 5K). These results suggest that BECN1 downregulates HSPA5 expression by binding to HSPA5, which in turn promotes the ubiquitination degradation of HMGCR and ultimately inhibits cholesterol production in OC cells.

Next, we further explored the regulatory role of ESM1 on the downstream BECN1/HSPA5/HMGCR axis. Co-IP results showed that ESM1 silencing promoted the binding of HSPA5 to BECN1, whereas it inhibited the binding of HSPA5 to HMGCR (Fig. 5L). In addition, we found that ESM1 overexpression inhibited the ubiquitination modification of HMGCR, and conversely, ESM1 silencing promoted the ubiquitination modification of HMGCR (Fig. 5M). These data illustrate that ESM1 inhibits the ubiquitination modification of HMGCR by promoting the binding of HSPA5 to HMGCR instead of BECN1.

### The expression of IGF2BP3 was correlated with cholesterol content in OC cells

**Fig. 6:**
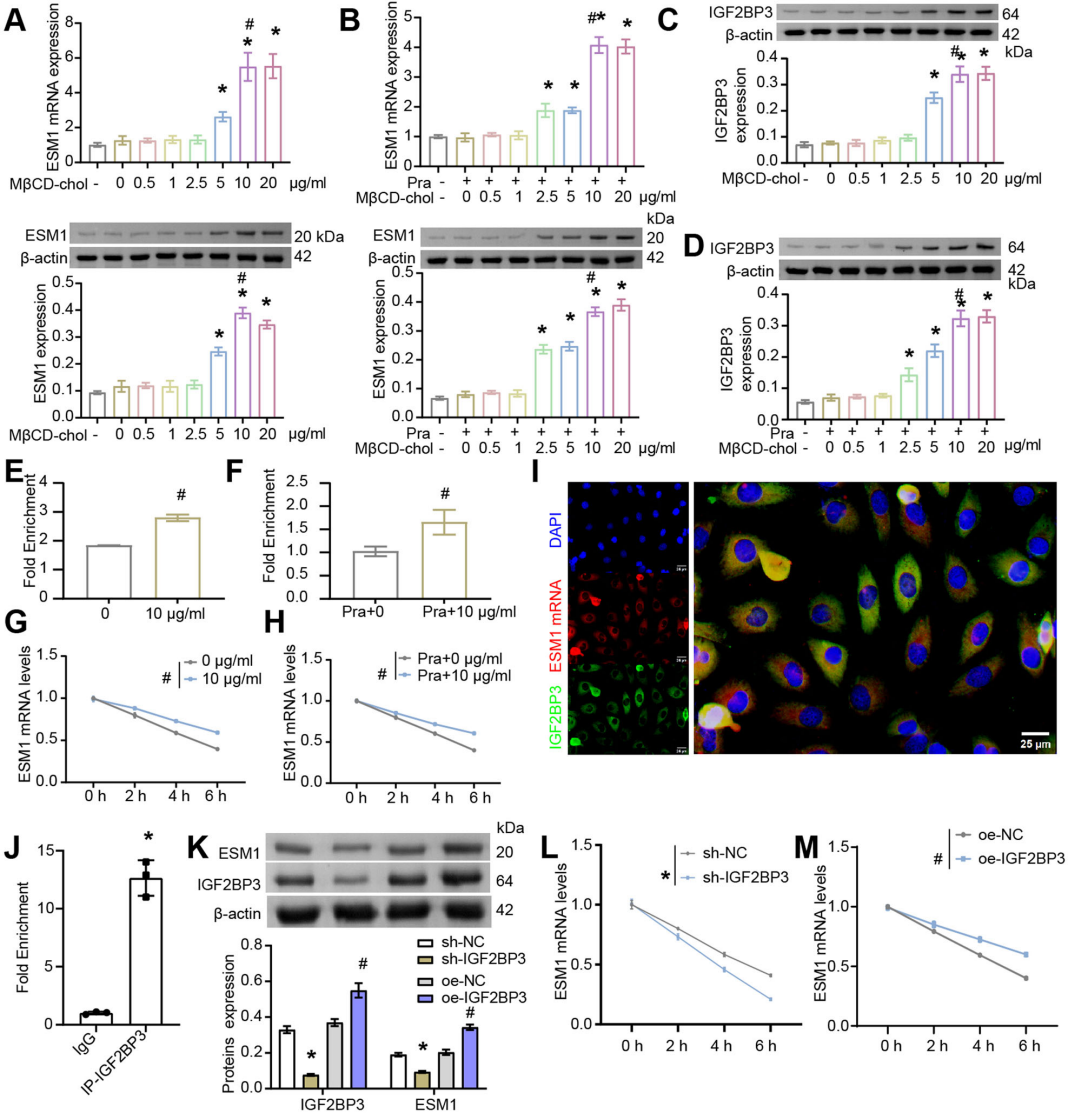
The expression of m6A demethylase IGF2BP3 was correlated with cholesterol content in SKOV3 cells as well as regulated the stability of ESM1 mRNA. **A**, **B** RT-qPCR and western blot analysis of ESM1 level in SKOV3 cells. **C**, **D** Western blot analysis of IGF2BP3 expression in OC cells. *p < 0.05 vs. 0 μg/mL MβCD-chol. ^#^*p* < 0.05 vs. 5 μg/mL MβCD-chol. **E**, **F** The m6A levels of ESM1 mRNA were evaluated using RIP-PCR. **G**, **H** The stability of ESM1 mRNA in OC cells was analyzed by actinomycin D treatment assay. ^#^*p* < 0.05 vs. 0 μg mL MβCD-chol or Pra+0 μg mL MβCD-chol. **I** The expressions of ESM1 mRNA and IGF2BP3 in OC cells were collocated by FISH and IF analysis. **J** The interaction between ESM1 mRNA and IGF2BP3 was verified by RIP-qPCR. *p < 0.05 vs. IgG. **K** Western blot analysis of IGF2BP3 and ESM1 levels in OC cells. **L**, **M** The stability of ESM1 mRNA in OC cells was analyzed by actinomycin D treatment assay. *p < 0.05 vs. sh-NC, ^#^*p* < 0.05 vs. oe-NC.

In OC, IGF2BP3 is highly expressed [42]. The study has found that cholesterol content is positively correlated with m6A reader IGF2BP3 level [43]. Based on the above analysis, we speculated that cholesterol promoted IGF2BP3 to some extent. To this end, we added different concentrations of exogenous cholesterol in the presence or absence of Pra to analyze the expression of ESM1 and IGF2BP3 and the stability of ESM1 mRNA. The results showed that exogenous cholesterol promoted the expressions of ESM1 mRNA and protein in OC cells in a concentration-dependent manner (Fig. 6A-B and S5A-B). In addition, exogenous cholesterol promoted the protein expression of IGF2BP3 in OC cells (Figs. 6C-D and S5C-D). Considering that IGF2BP3 is a key m6A reader protein, we next examined the m6A level of ESM1 mRNA. As shown in Figs. 6E-F and S5E-F, exogenous cholesterol significantly increased the m6A level of ESM1 mRNA with or without the presence of Pra. Furthermore, the results of the actinomycin D treatment assay displayed that the content of ESM1 mRNA decreased with the extension of time. Exogenous cholesterol intervention significantly delayed the degradation of ESM1 mRNA in OC cells (Figs. 6G-H and S5G-H). In general, these results proved that the expression of m6A reader IGF2BP3 was correlated with cholesterol content in OC cells.

### The m6A reader IGF2BP3 regulates ESM1 mRNA stability

Online prediction by RMBase2.0 (https://rna.sysu.edu.cn/rmbase/) revealed that ESM1 mRNA may undergo m6A methylation modification, and the results in Figs. 6E-F and S5E-F have demonstrated that exogenous cholesterol raises the m6A level of ESM1 mRNA. Through catRAPID (http://s.tartaglialab.com/page/catrapid_group) online prediction, it was found that ESM1 might bind to m6A reader IGF2BP3. FISH and IF analysis confirmed the co-expression of ESM1 mRNA and IGF2BP3 (Figs. 6I and S5I). RIP-qPCR further verified the interaction between ESM1 mRNA and IGF2BP3 in OC cells (Figs. 6J and S5J). To further verify the impact of m6A reader IGF2BP3 on the level of ESM1, IGF2BP3 expression in OC cells was silenced or overexpressed. Down-regulation or up-regulation of IGF2BP3 expression in OC cells validated the efficiency of IGF2BP3 silencing or overexpression. In addition, IGF2BP3 silencing inhibited ESM1 protein expression and accelerated the degradation of ESM1 mRNA. However, overexpression of IGF2BP3 reverses this situation (Figs. 6K-M and S5K-M). Together, these results suggested that the IGF2BP3 promoted the stability of ESM1 mRNA.

### ESM1 modified by m6A methylation regulated lipid metabolism in OC in vivo

**Fig. 7:**
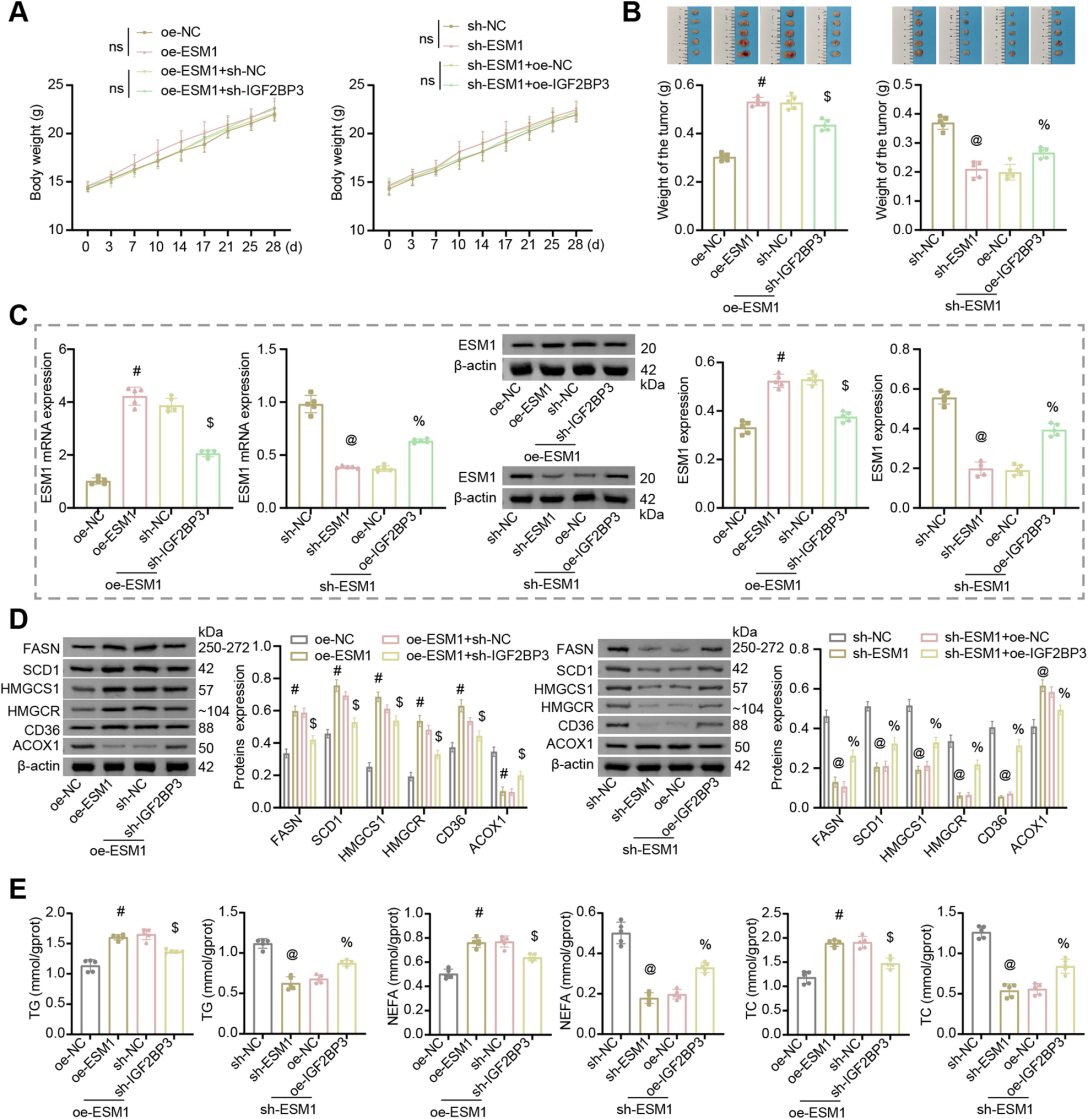
ESM1 modified by m6A methylation regulated lipid metabolism in OC mice. **A** Growth curve of nude mice. **B** The weight of the tumor. **C** RT-qPCR and western blot analysis of ESM1 level in tumor tissues. **D** Western blot analysis of FASN, SCD1, HMGCS1, HMGCR, CD36, and ACOX1 levels in tumor tissues. **E** Biochemical kits were utilized to assess the contents of TG, NEFA, and TC in the serum. ^#^*p* < 0.05 vs. oe-NC, ^$^*p* < 0.05 vs. oe-ESM1+sh-NC, ^@^*p* < 0.05 vs. sh-NC, ^%^*p* < 0.05 vs. sh-ESM1+oe-NC.

Based on the above findings, we conducted animal experiments to further explore the in vivo regulation of m6A methylated ESM1 on lipid metabolism in OC. A2780 cells stably transfected with oe-ESM1, sh-ESM1, sh-IGF2BP3, and oe-IGF2BP3 were injected into nude mice to observe the tumor growth. The body weight of nude mice in each group increased gradually with time, but there was no significant difference between the groups (Fig. 7A). The silencing of IGF2BP3 inhibited the promotion of tumor growth by ESM1 overexpression, while the overexpression of IGF2BP3 reversed the inhibition of ESM1 silencing on tumor growth (Fig. 7B). Furthermore, silencing of IGF2BP3 inhibited the expression of ESM1 in the tumor, while overexpression of IGF2BP3 promoted the expression of ESM1 in the tumor (Fig. 7C). Subsequently, by analyzing the levels of relevant indexes of lipid metabolism, it was found that silencing of IGF2BP3 inhibited the up-regulation of oe-ESM1 on FASN, SCD1, HMGCS1, HMGCR, and CD36 expressions, the down-regulation of ACOX1 expression, and the promotion of TG, NEFA and TC accumulation in peripheral blood. The overexpression of IGF2BP3 reversed the down-regulation of FASN, SCD1, HMGCS1, HMGCR, and CD36 expression, up-regulation of ACOX1 expression, and inhibition of TG, NEFA, and TC accumulation in peripheral blood by ESM1 silencing (Fig. 7D-E). Together, these results suggested that ESM1 modified by m6A methylation regulated lipid metabolism in OC in vivo.

### Clinical validation of IGF2BP3/ESM1/KLF10/BECN1 axis in OC tissues

To investigate the importance of ESM1 in human OC patients, we collected tumor tissues from 94 patients diagnosed with OC and further statistically analyzed the correlation between the expression level of ESM1 and the clinical and pathological characteristics of patients. The results displayed that there was no significant correlation between the expression level of ESM1 and the age, clinical stage, and methods of treatment of OC patients (Table 3). Furthermore, levels of ESM1, IGF2BP3, KLF10, BECN1, HMGCR, and SCD1 were analyzed by multiplex IF. The best cut-off point was obtained through X-tile to classify OC patients into high/low expression groups [44]. The results indicated that the expression of ESM1 (*p* = 0.0109), HMGCR (*p* = 0.0434), and SCD1 (*p* = 0.0299) was significantly correlated with the survival period of OC patients. Specifically, OC patients with high expression levels of ESM1, HMGCR, and SCD1 had shorter survival periods (Fig. S6A). Additionally, in OC patients with high expression of IGF2BP3 or SCD1, patients with low expression of ESM1 had longer survival periods than those with high expression of ESM1 (Fig. S6B). The above results proved that in OC patients, ESM1 indeed affected the prognosis of OC patients by regulating the IGF2BP3/ESM1/KLF10/BECN1 axis and could be a prognostic indicator.

## Discussion

Of all gynecological tumors, OC is the deadliest known. Despite extensive research in this area, OC still shows high incidence, high recurrence rates, and low survival rates. The lack of promising early detection tools is one of the major challenges associated with poor survival in patients with OC [45]. Here, we identified ESM1 as a carcinogenic factor in OC. ESM1 regulates lipid metabolism in OC through IGF2BP3/ESM1/KLF10/BECN1 positive feedback, thus promoting tumor growth and development. ESM1 was expected to be an effective biomarker for the diagnosis and treatment of early OC.

Dysregulation of ESM1 is widespread in various malignant tumors. The pan-cancer analysis found that ESM1 is overexpressed in multiple cancer types. High ESM1 levels are associated with shorter overall survival and disease-free survival [46]. The study has shown that knocking down ESM1 expression reduces the proliferation and migration activity of head and neck cancer cells [47]. In our previous studies, ESM1 was a key secreted protein that promoted OC cell proliferation, apoptotic escape, and angiogenesis [10]. Here, ESM1 silencing significantly reduced the proliferative activity and tumorigenicity of OC cells in vivo. Moreover, it was intracellular ESM1 that exerted regulatory effects, rather than secretory ESM1. This suggested that cancer inhibition could be achieved by regulating ESM1 expression, and ESM1 could be regarded as a potential therapeutic target for OC.

The study has shown that alterations in lipid metabolism can support the high energy requirements of cancer cells and evade anti-tumor immune responses. Lipid metabolism disorders are often related to drug resistance and poor prognosis [21]. The research has shown that many molecules are involved in lipid metabolism regulation. For example, FASN, a key enzyme in the de novo synthesis of fatty acids, is associated with tumor-related signaling pathways [48]. SCD1 can regulate the ratio of monounsaturated fatty acids (MUFA) to saturated fatty acids (SFA) [49]. Here, ESM1 silencing improved lipid metabolism disorder in OC cells, which was manifested as down-regulated levels of FASN, SCD1, HMGCS1, HMGCR, CD36, TG, TC and NEFA, and up-regulated levels of ACOX1. This suggested that ESM1 influenced lipid metabolism in OC by regulating the expression of these molecules.

The previous study has shown that is a promising anticancer strategy to target altered lipid metabolic pathways [13]. Targeting SRP-1-driven lipid metabolism can treat glioblastoma [50]. In addition, SCAP/SREBP can serve as a promising lipid metabolic target for cancer therapy [51]. Here, mRNA sequencing results showed that BECN1, a gene associated with autophagy, might be related to ESM1 regulation of lipid metabolism in OC. Subsequent BECN1 overexpression and CQ intervention results confirmed that ESM1 indeed inhibited lipolysis in OC by blocking BECN1-mediated autophagy. However, BECN1 expression is regulated by the transcription factor KLF10. An important relationship between KLF10 and cell proliferation, apoptosis, glucose, and lipid metabolism has been confirmed [52]. In addition, KLF10 is a tumor suppressor gene [53]. These results suggested that ESM1/KLF10/BECN1 could be a potential lipid metabolic target for OC treatment. In further studies, we explored the specific mechanism by which ESM1 blocks BECN1-mediated autophagy inhibition of lipolysis in OC. The results showed that the competitive combination of BECN1 and HSPA5 promoted the ubiquitination of HMGCR and inhibited the expression of HMGCR. When the endoplasmic reticulum stress (ERS) occurs, HSPA5 translocates to the cell surface, mitochondria, and nucleus, where it can combine with proteins to perform functions. HSPA5 can play different functional roles in cell viability and immune regulation, resulting in various diseases, including cancer [54]. In the related study, SIAH1 ubiquitination-modified HMGCR inhibited lung cancer progression and increased drug sensitivity by regulating cholesterol synthesis [55]. These results verified that ESM1 could promote the development of OC by regulating lipid metabolism through BECN1/HSPA5/HMGCR axis.

The m6A RNA methylation is involved in the regulation of physiological and pathological processes, including cancer [24]. m6A modification and its regulators have the potential to be used as diagnostic markers and therapeutic targets for OC [56]. As an m6A reader, IGF2BP3 participates in regulating the mRNA stability of many tumor-related genes [26]. Here, exogenous cholesterol intervention showed that IGF2BP3 expression increased in OC cells, while ESM1 mRNA stability and protein expression decreased. In the related study, disrupting the stability of IGF2BP3-targeted mRNA can inhibit the progression of OC [27]. In further experiments, through IGF2BP3 overexpression or silencing intervention in vivo and in vitro, we confirmed that IGF2BP3 regulated ESM1 mRNA stability to affect lipid metabolism in OC.

This study successfully elucidated the specific mechanism of oncogene ESM1 regulating lipid metabolism in OC and provided some help for the development of therapeutic methods for OC. However, in the mechanism pathway, we only explored one m6A reader, IGF2BP3, and one autophagy gene, BECN1. In future work, more factors should be included to enrich the regulatory network of OC. In addition, the exact regulatory mechanism between the three proteins, BECN1/HSPA5/HMGCR, still needs further in-depth study.

## Conclusion

In summary, our results suggested that ESM1 regulated lipid metabolism in OC through positive feedback of IGF2BP3/ESM1/KLF10/BECN1. ESM1 was expected to be a prognostic biomarker and an effective therapeutic target for OC. This work will provide new insights into the development of novel OC treatment strategies based on ESM1 regulation.

## Supplementary information

Supplemental Material

Supplementary Information

## Data Availability

The datasets presented in this study can be found in online repositories. The names of the repository/repositories and accession number(s) can be found as follows: https://www.ncbi.nlm.nih.gov/sra/PRJNA1082038.
