# Supplementary material for: IGF2BP3/ESM1/KLF10/BECN1 positive feedback loop: a novel therapeutic target in ovarian cancer via lipid metabolism reprogramming
Source: Cell Death Dis. 2025 Apr 17;16(1):308. doi: 10.1038/s41419-025-07571-7 (PMC12003649; doi:10.1038/s41419-025-07571-7)
Supplement: Supplementary file 1 — Supplemental Material [file 41419_2025_7571_MOESM1_ESM.pdf]

Figure 1A

A2780

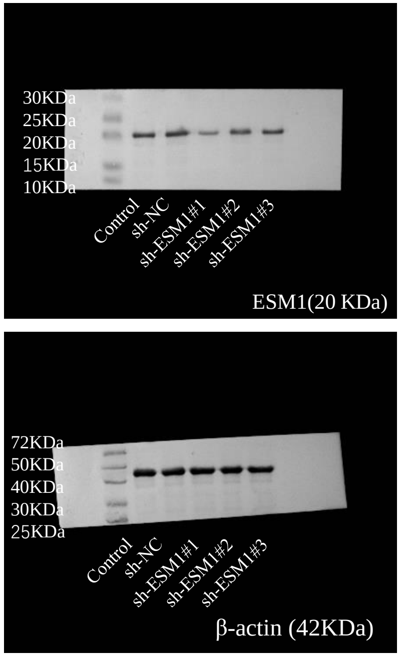

SKOV3

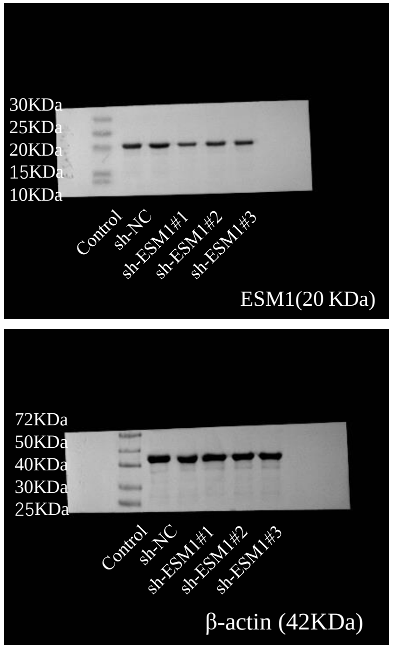

Figure 1C

A2780

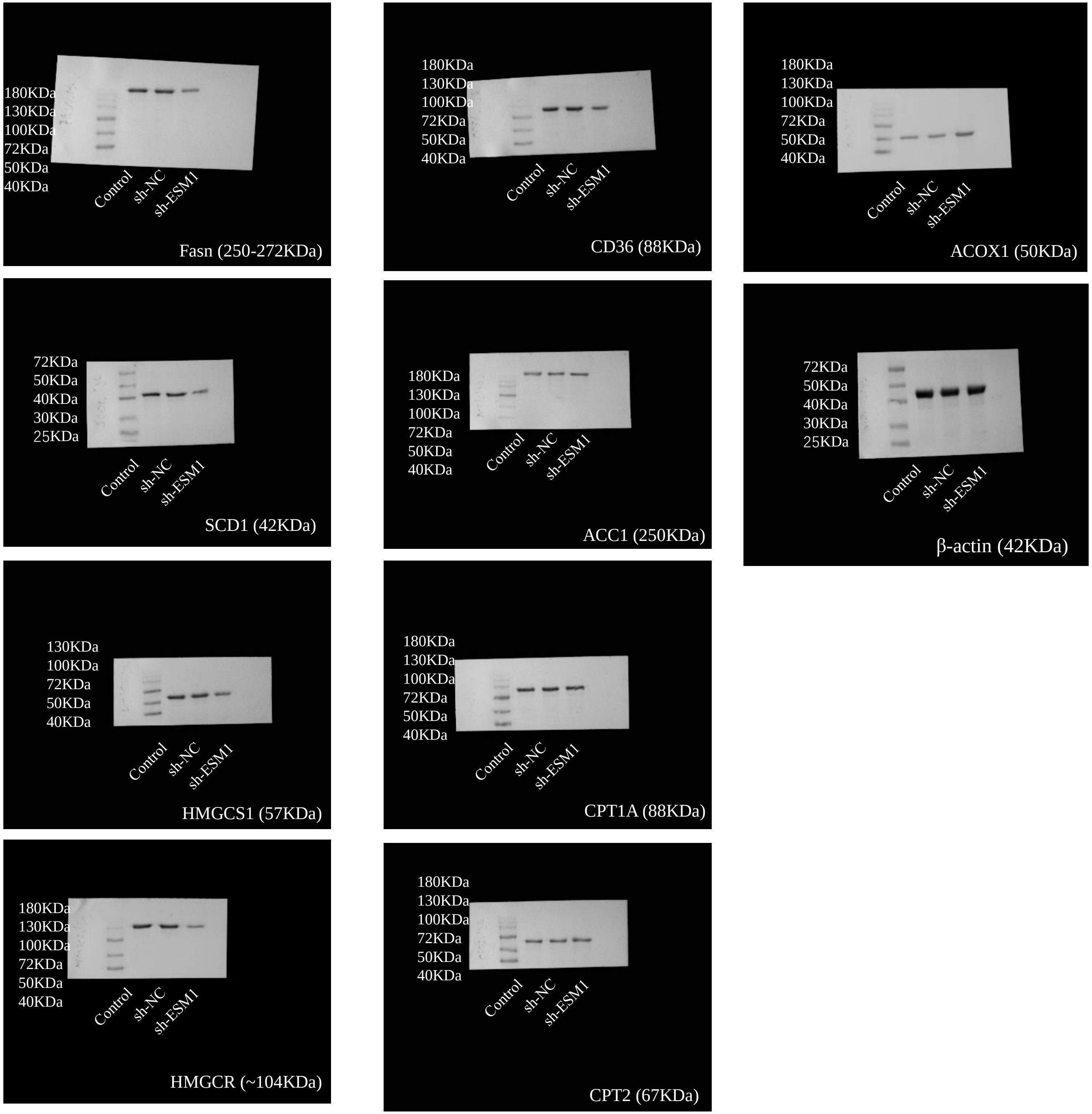

Figure 1C

SKOV3

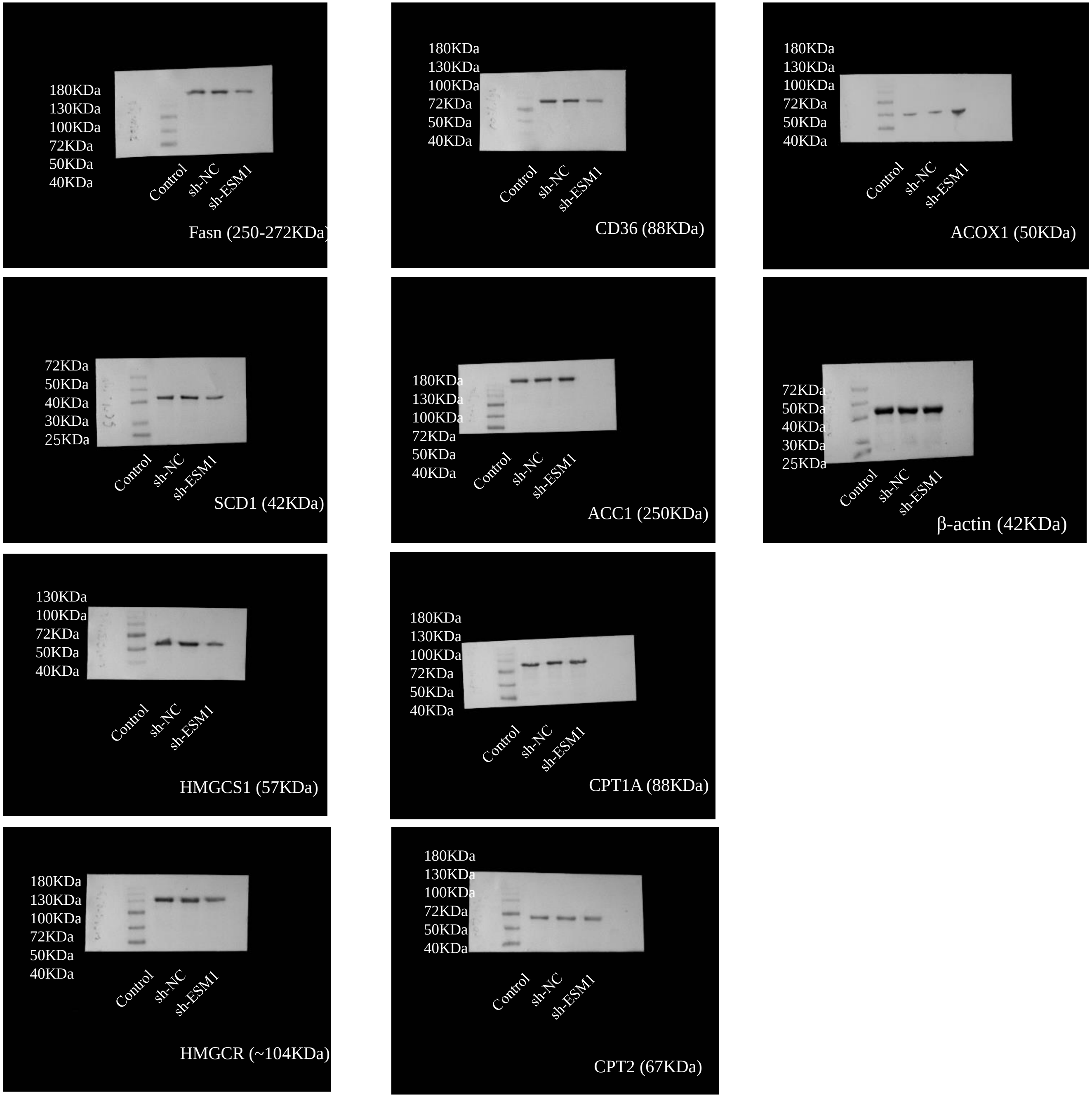

Figure 1D

A2780

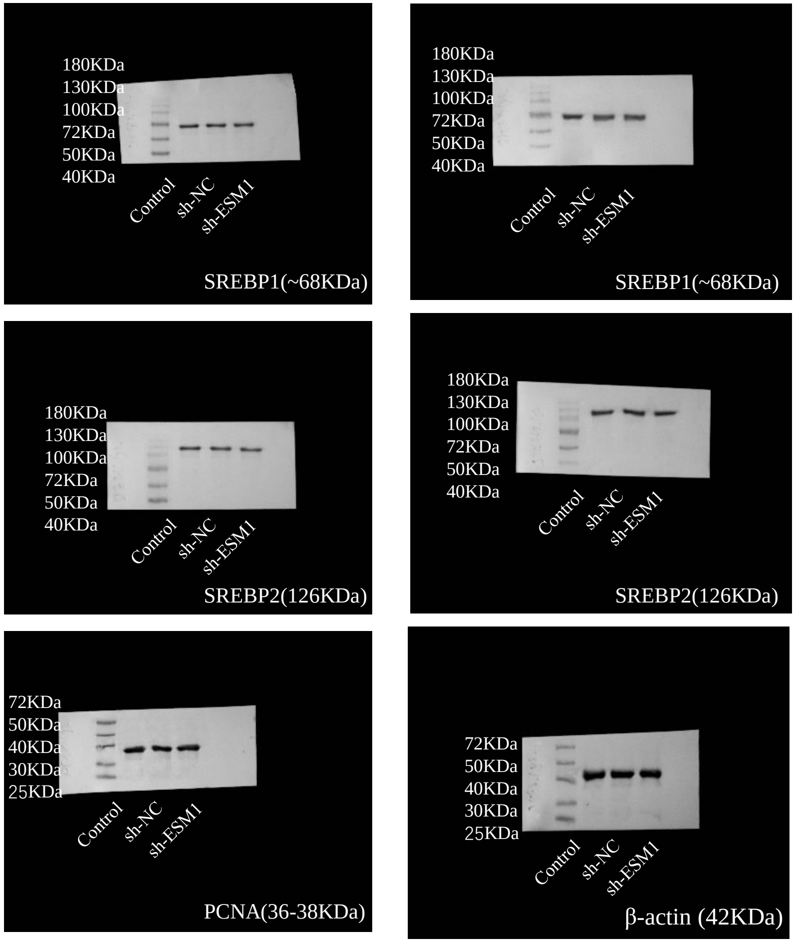

SKOV3

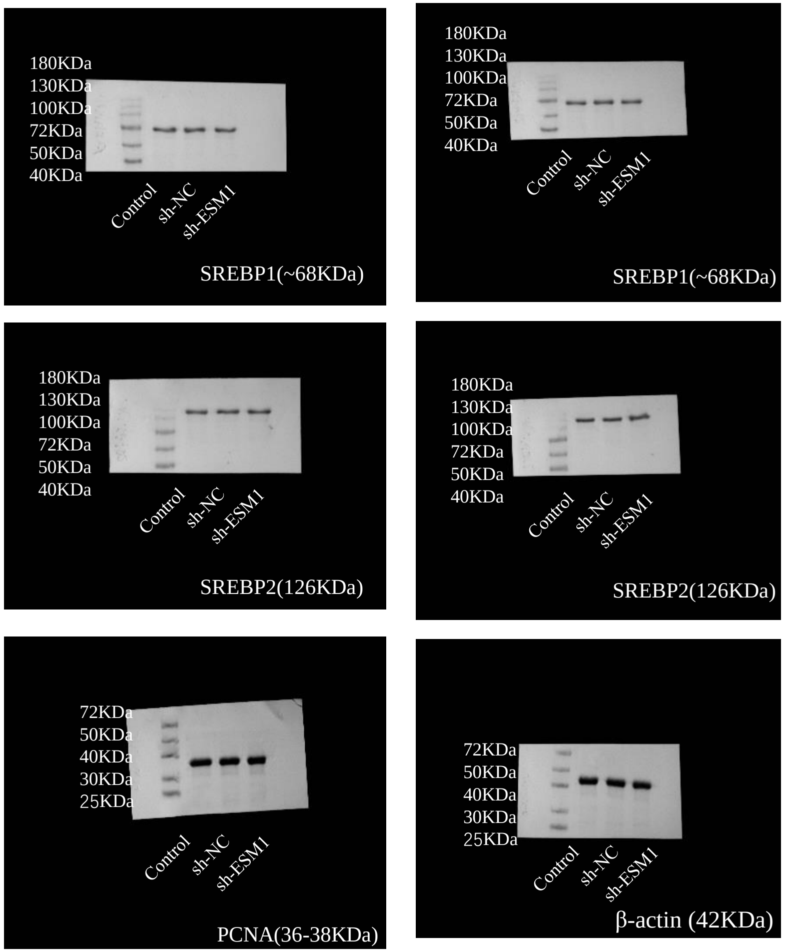

Figure 2C

A2780

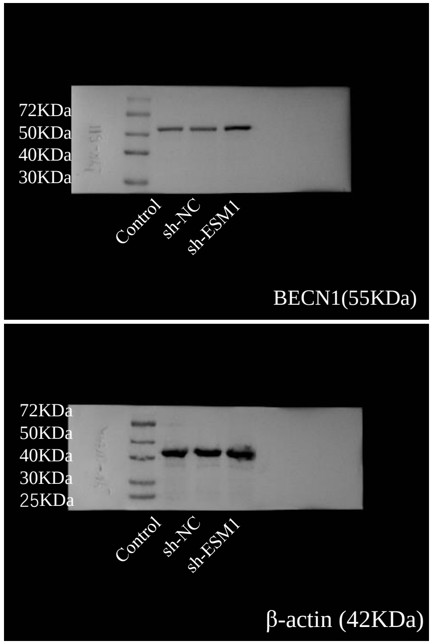

SKOV3

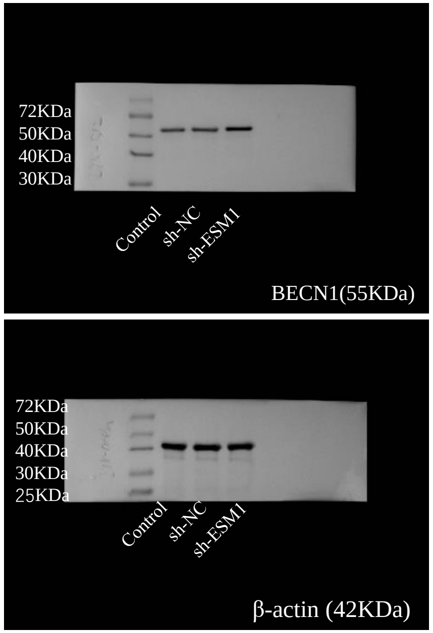

Figure 2F

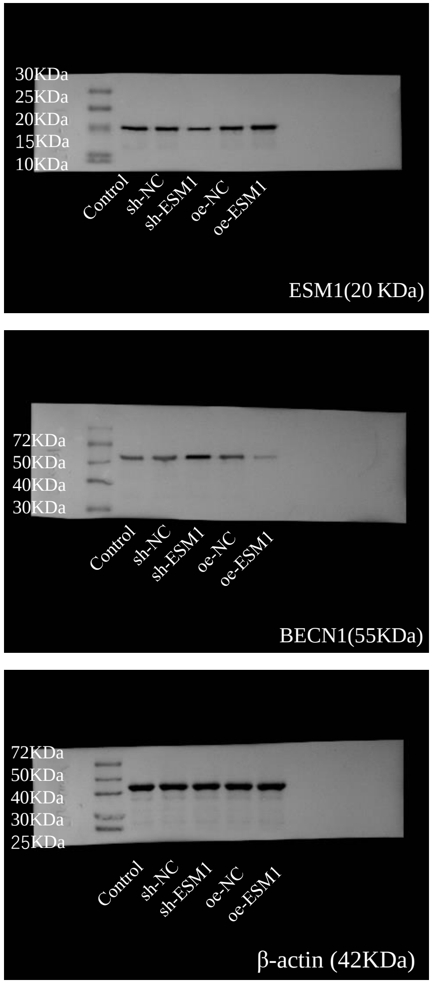

Figure 2G

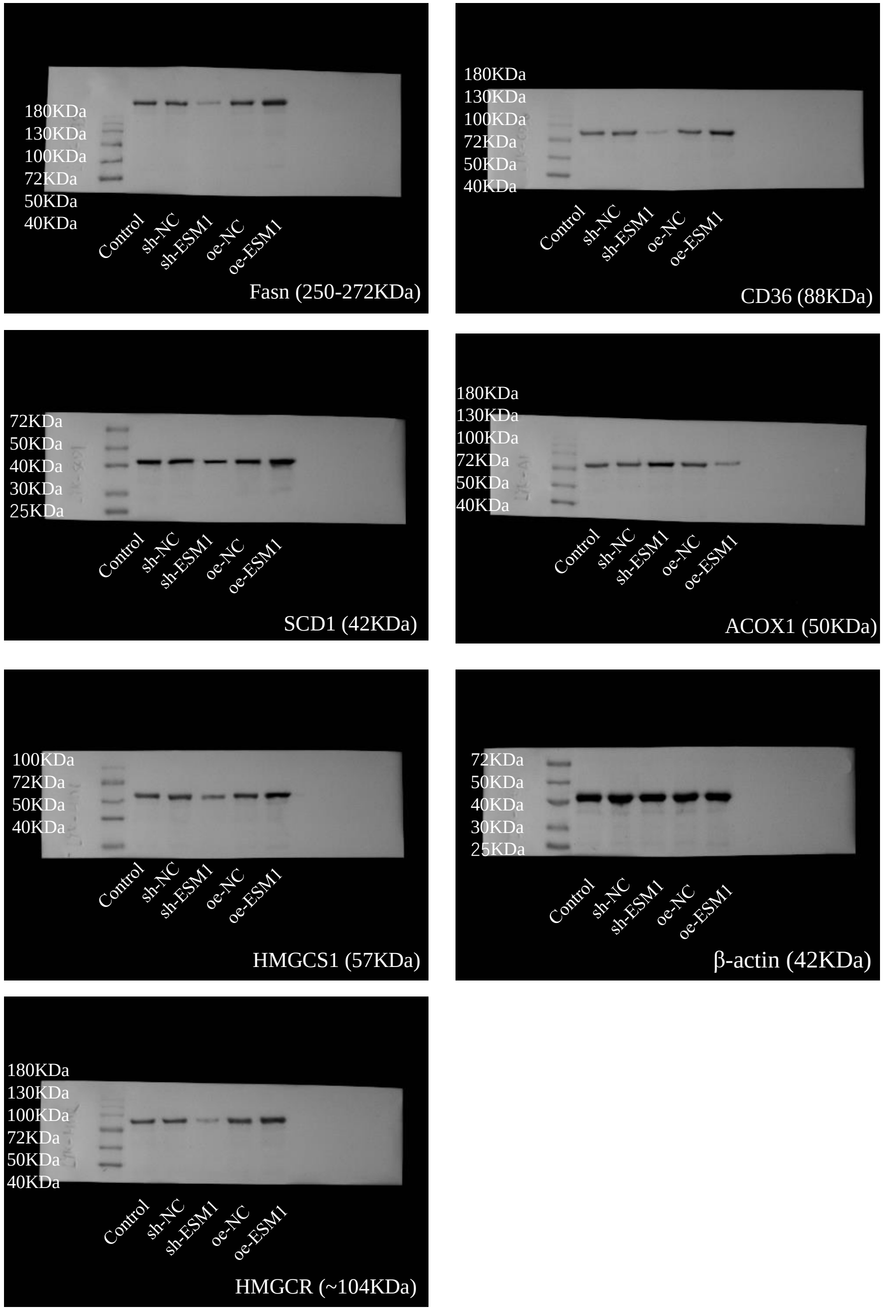

Figure 3A

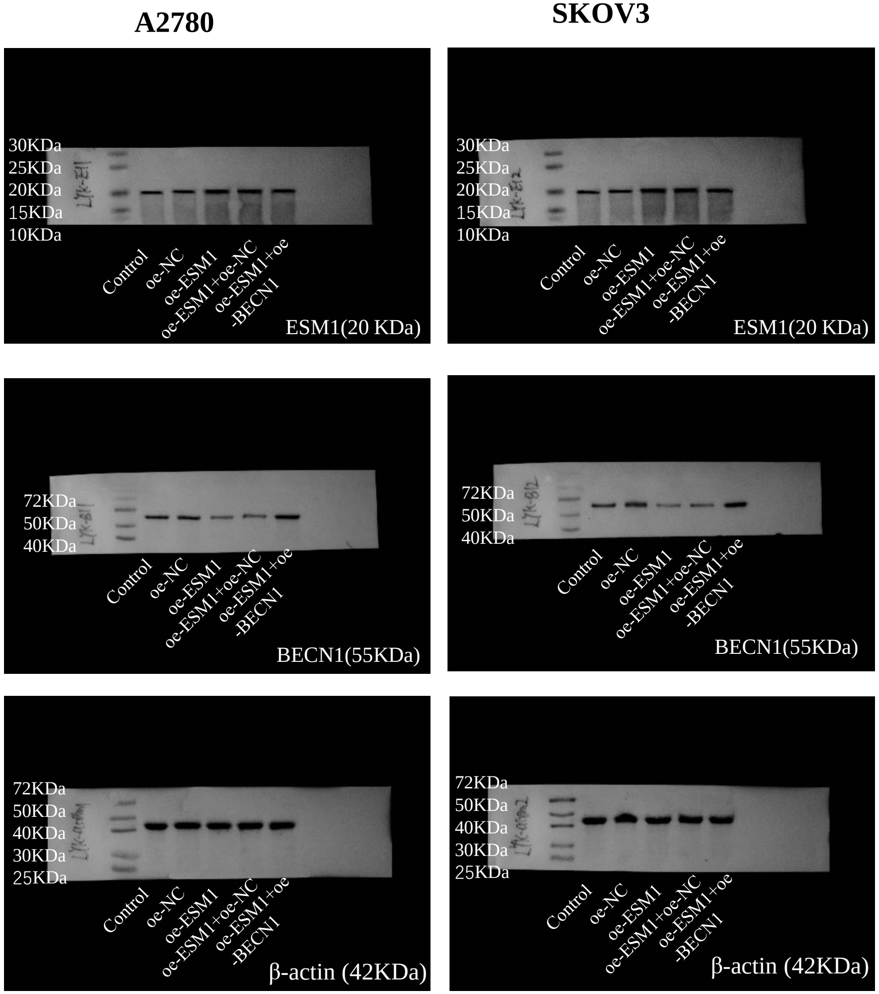

Figure 3B

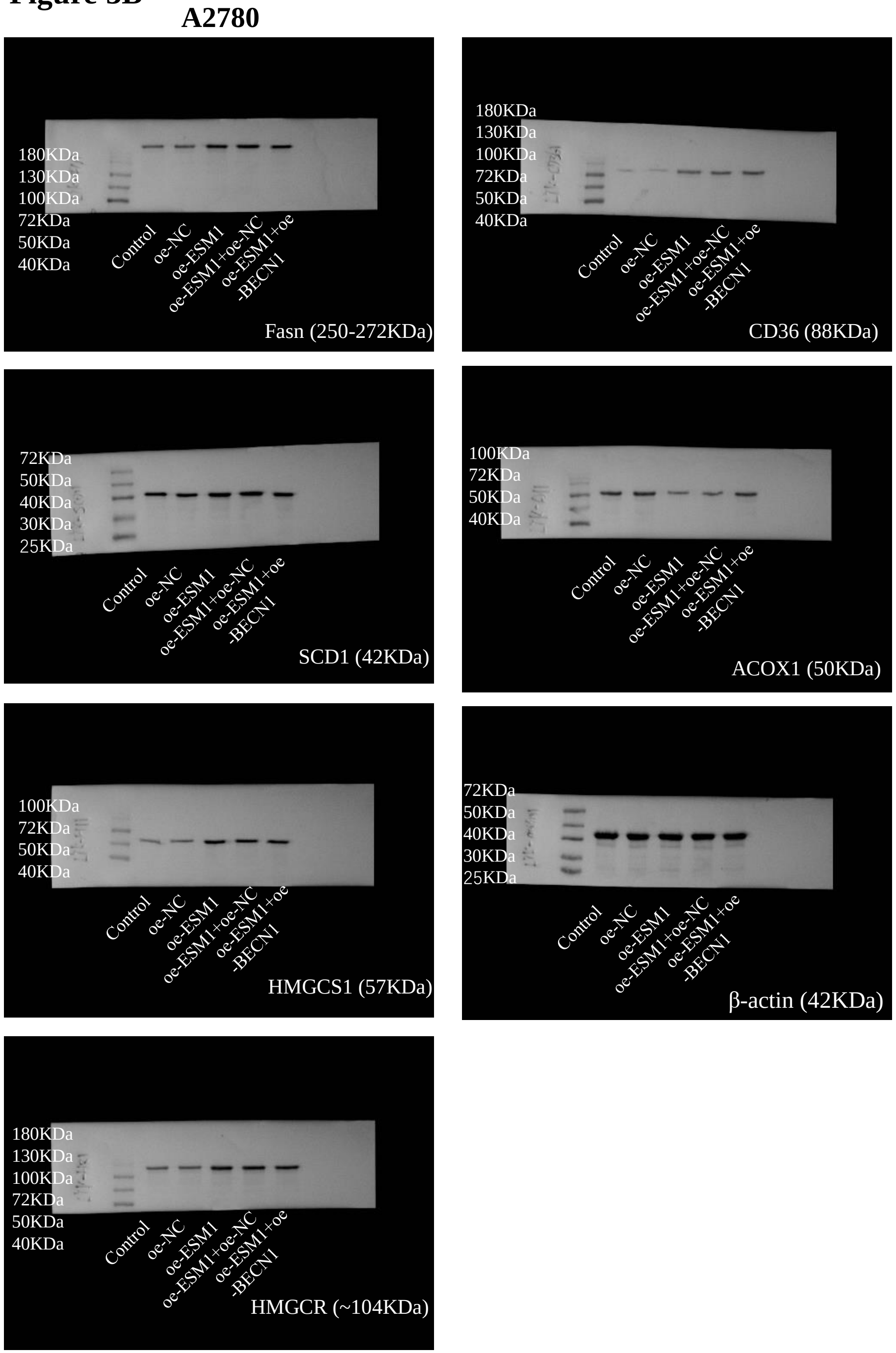

Figure 3B

SKOV3

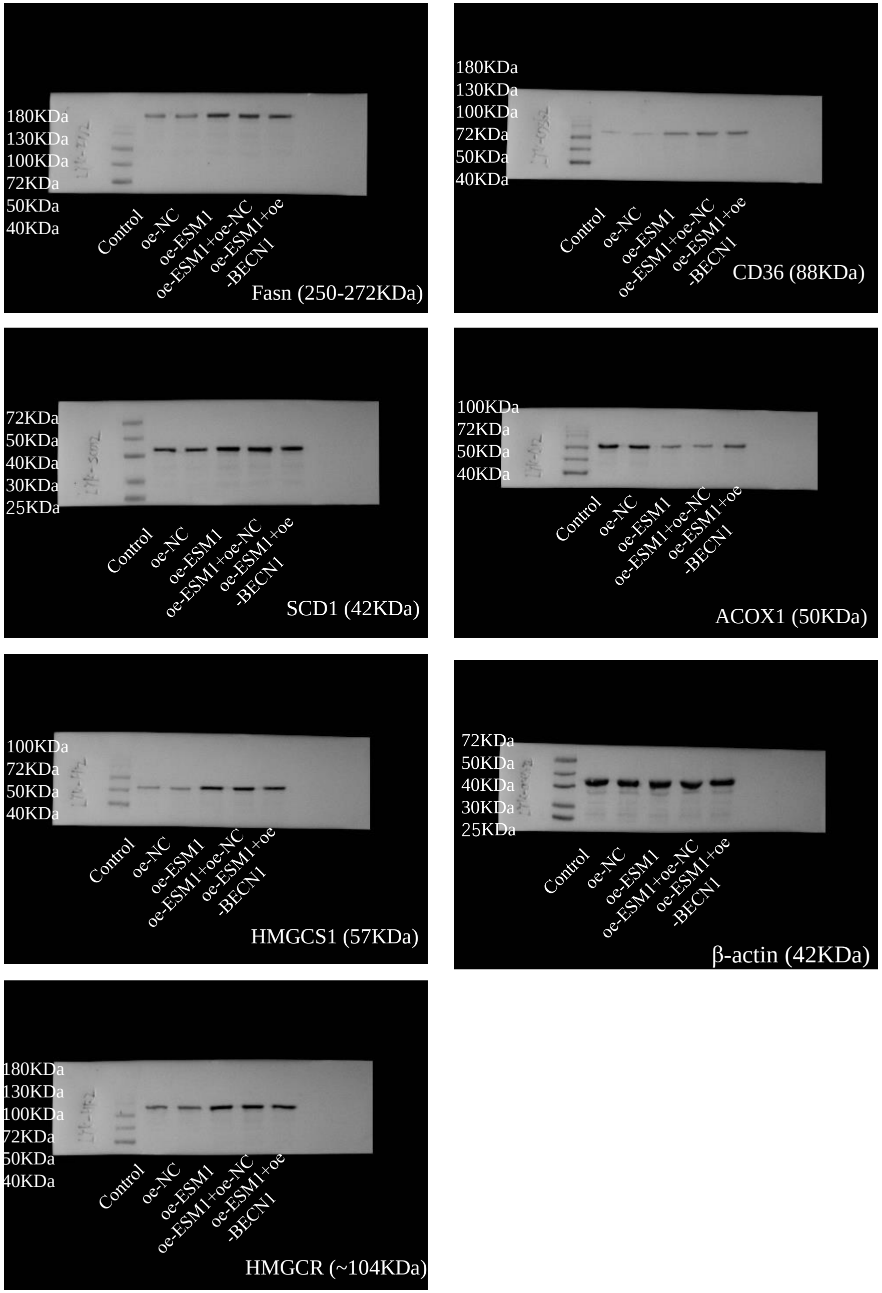

Figure 4A

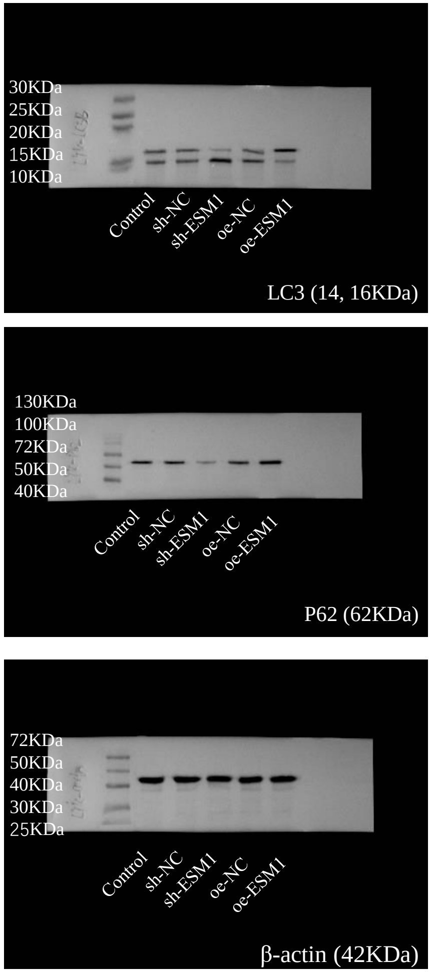

Figure 4B

A2780

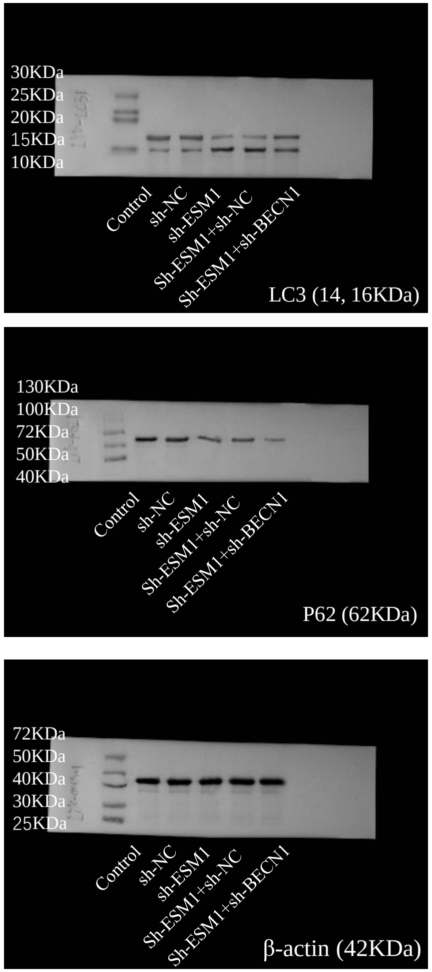

Figure 4C

Figure 4B

SKOV3

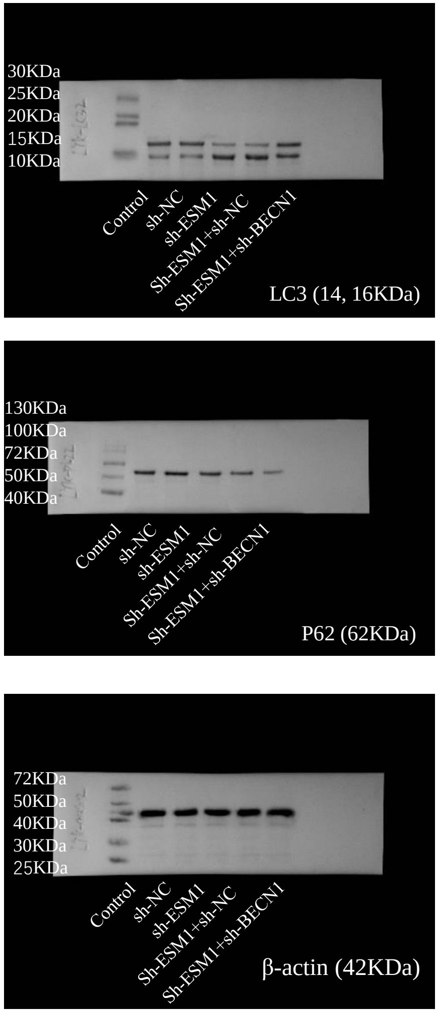

A2780 SKOV3

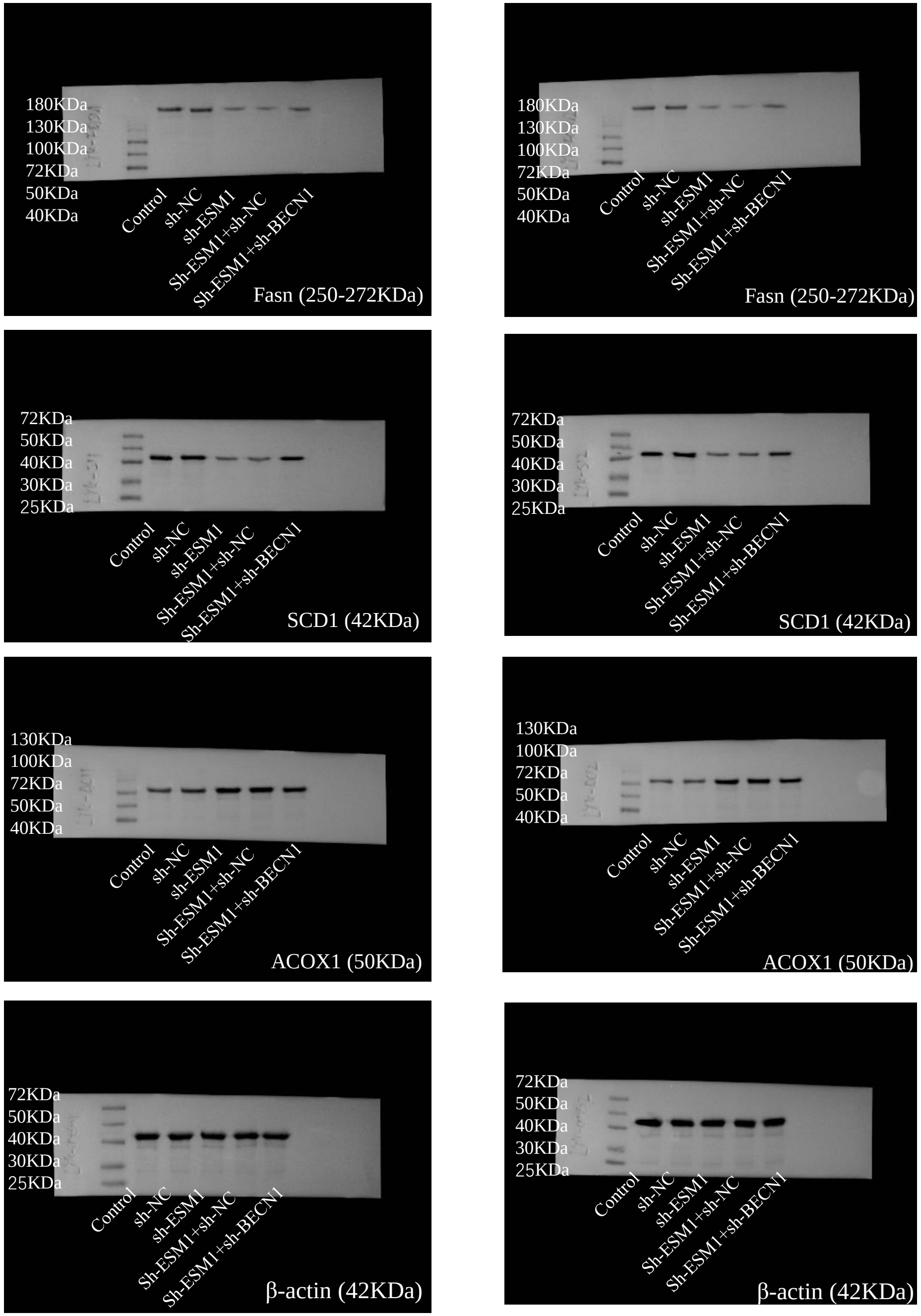

Figure 5A

A2780 **SKOV3**

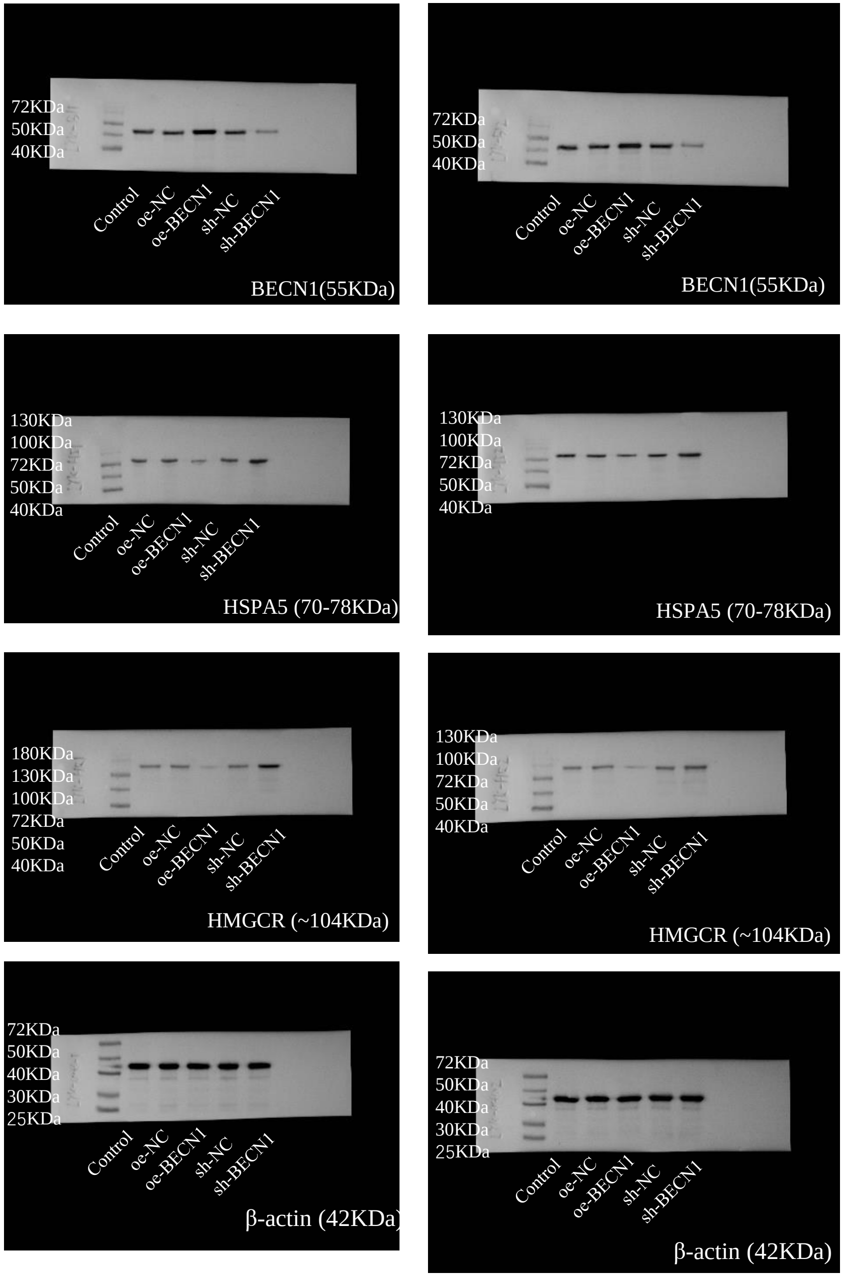

Figure 5C

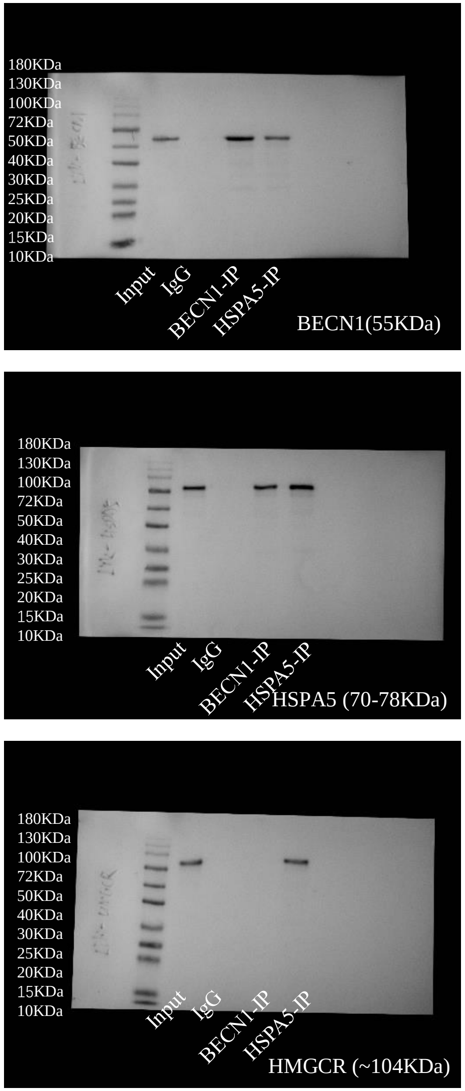

Figure 5D

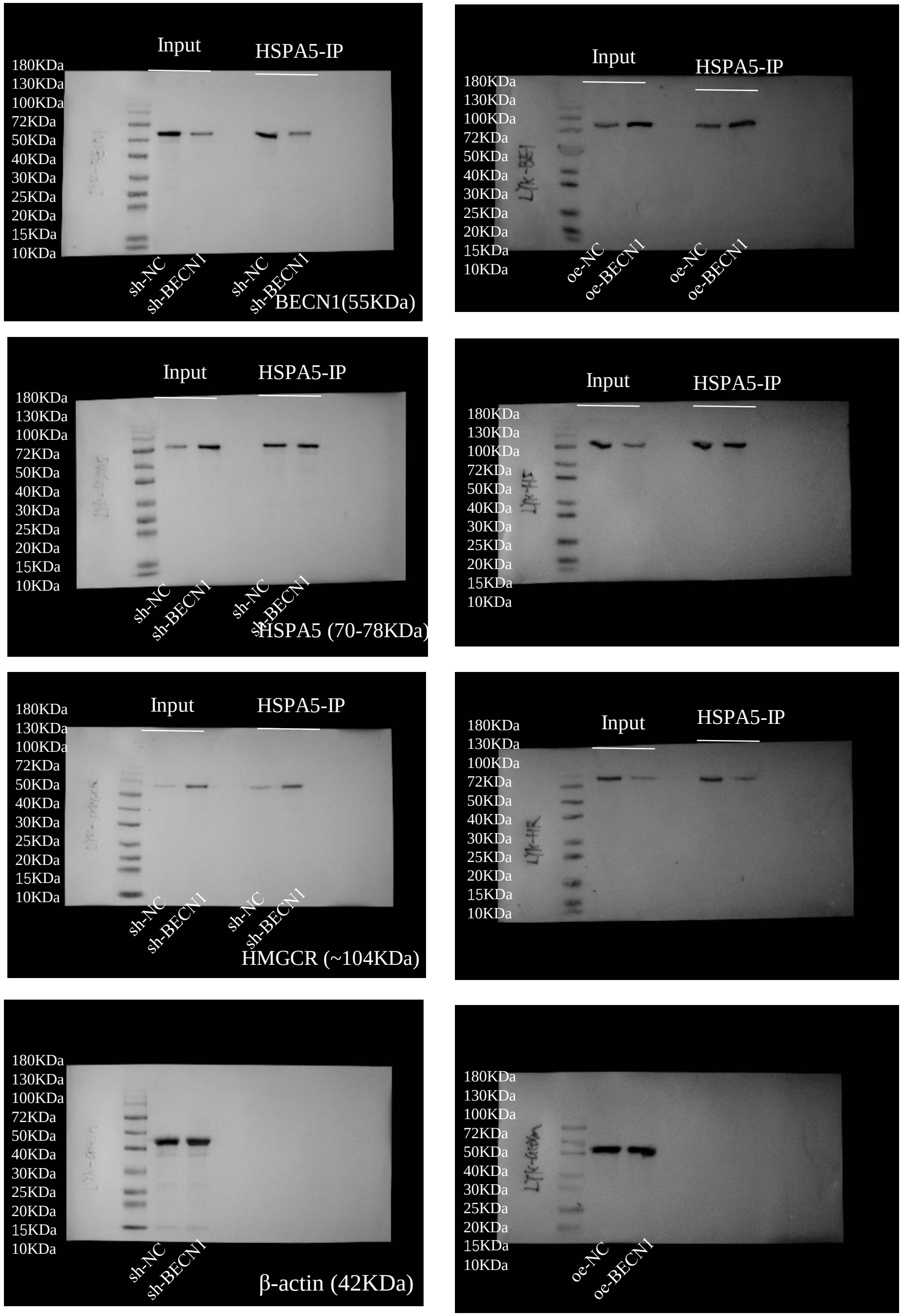

Figure 5E

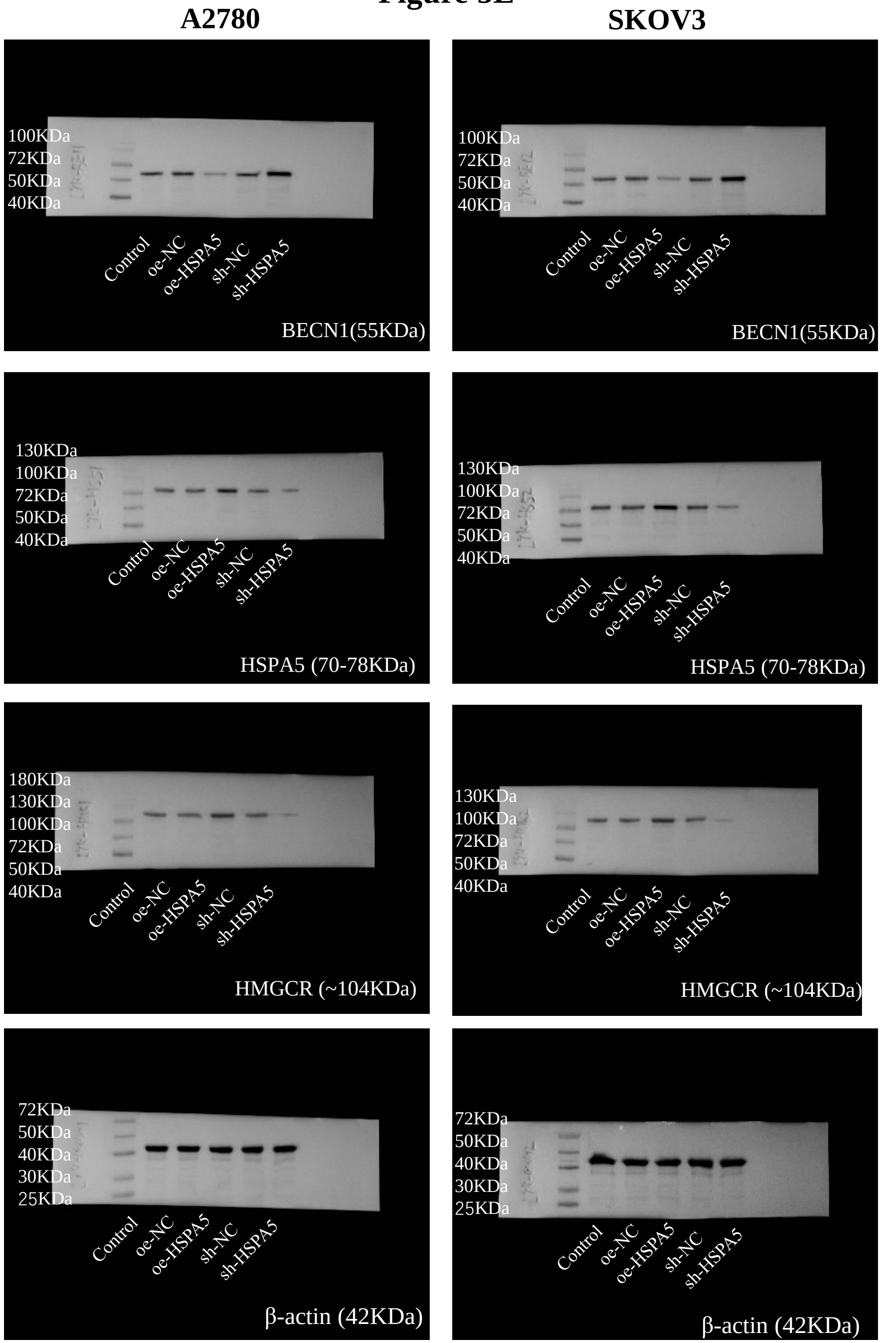

Figure 5F

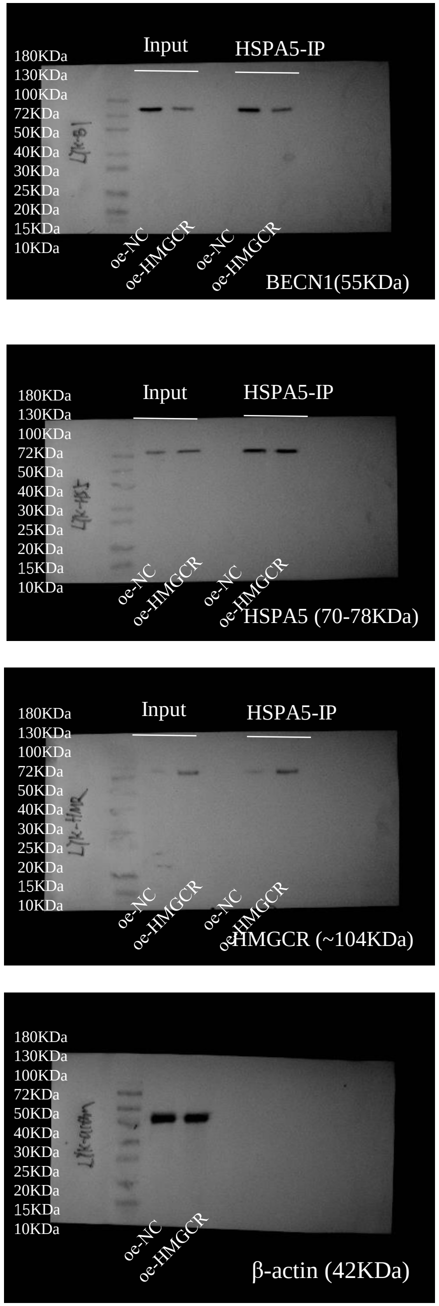

Figure 5H

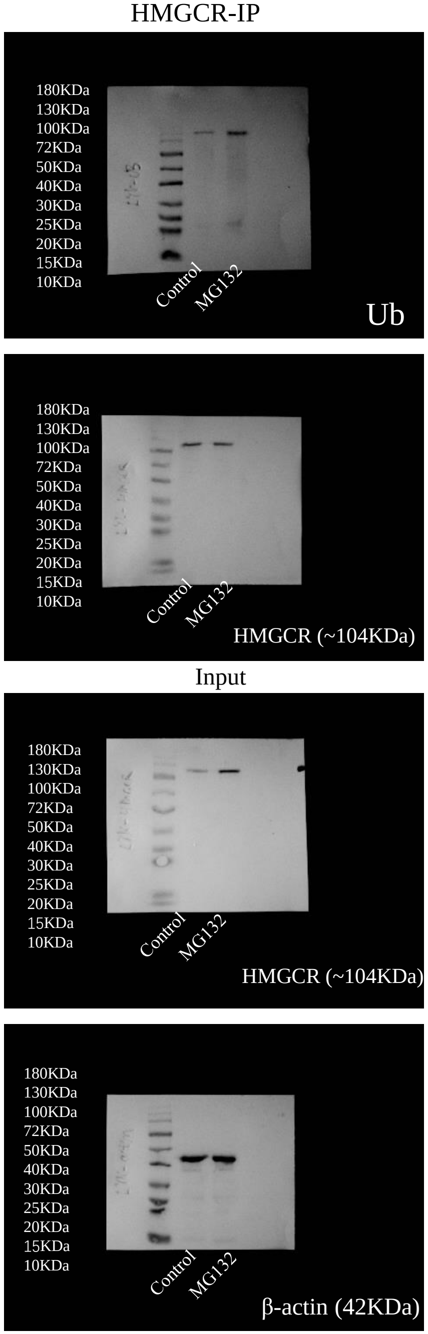

Figure 5I

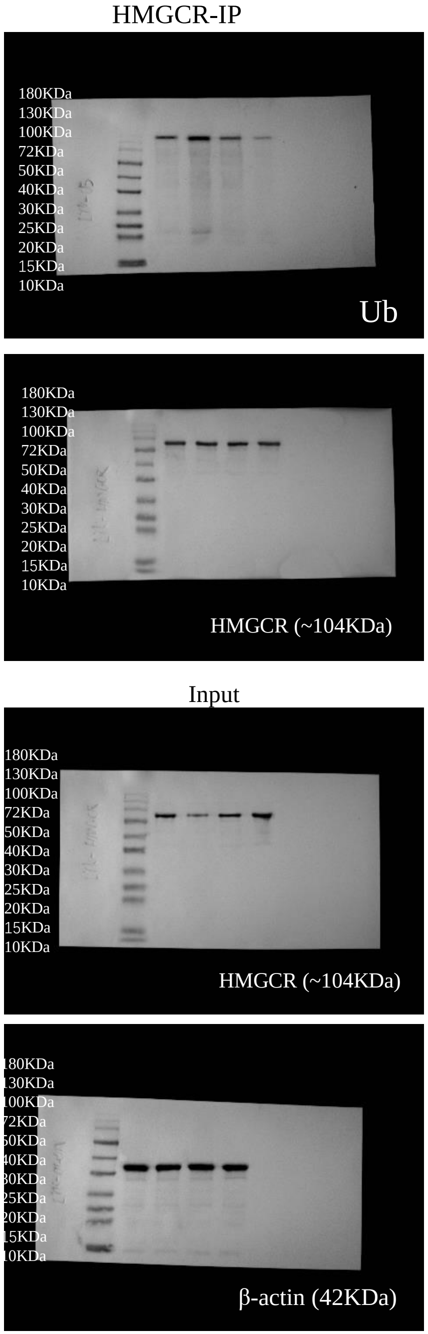

Figure 5J

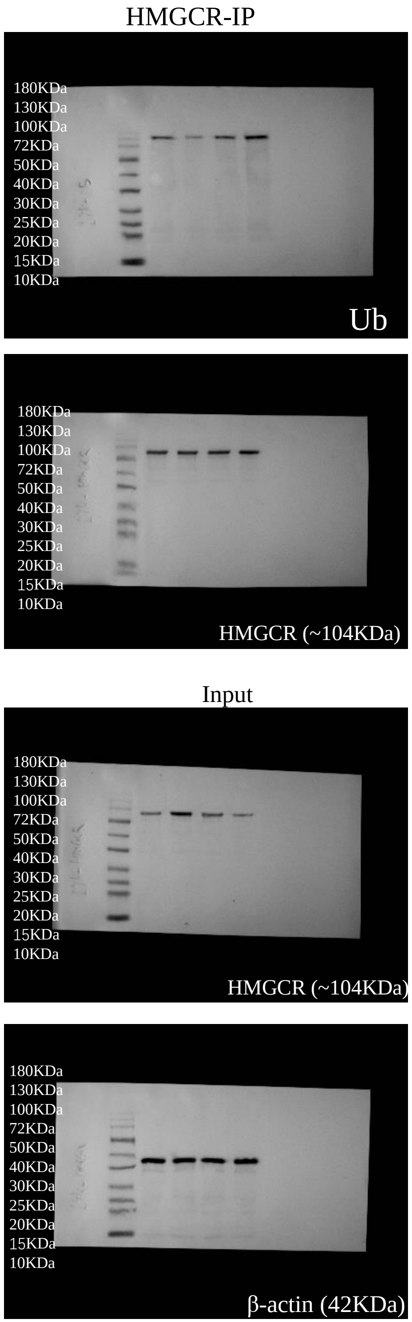

Figure 5K

A2780 SKOV3

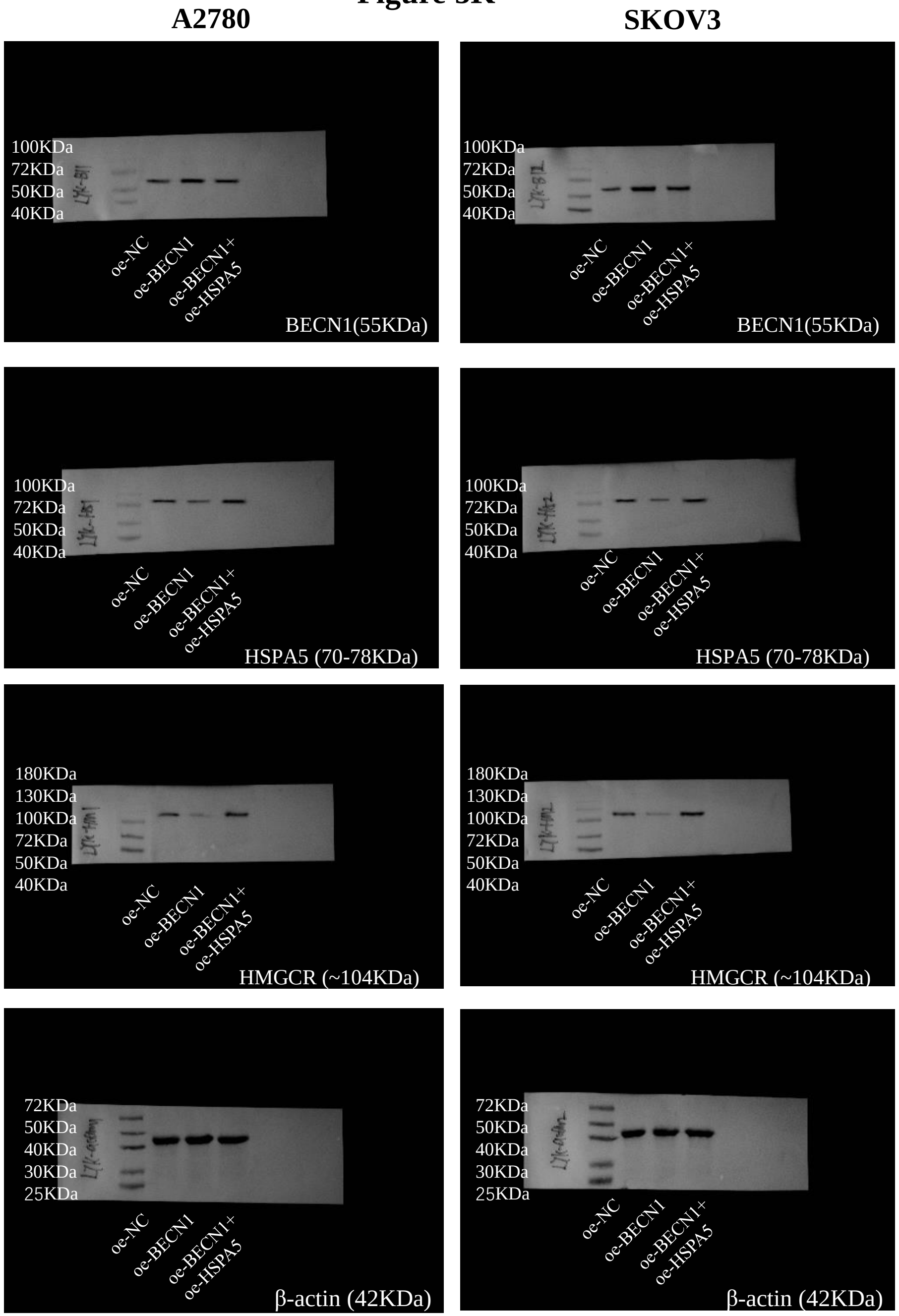

Figure 5L

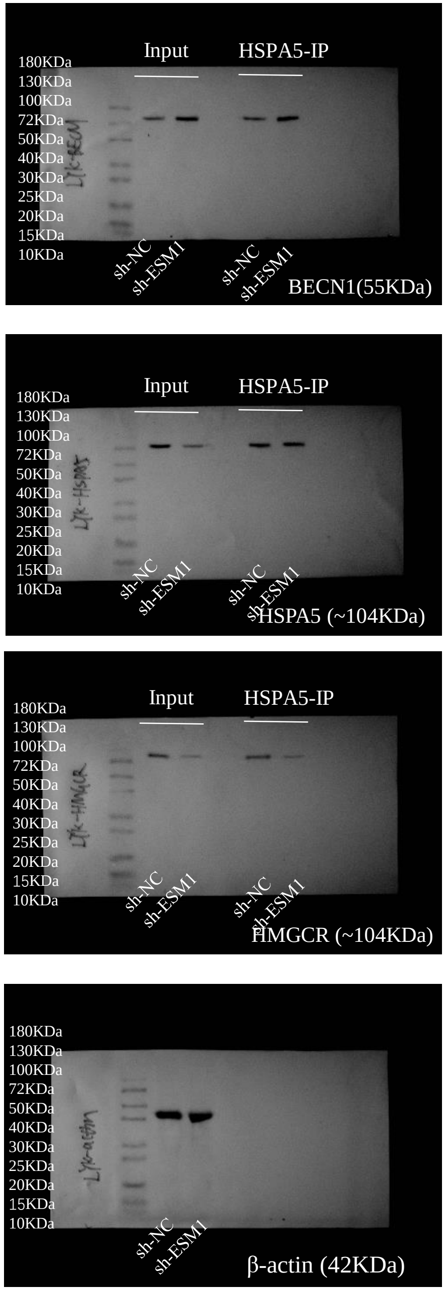

Figure 5M

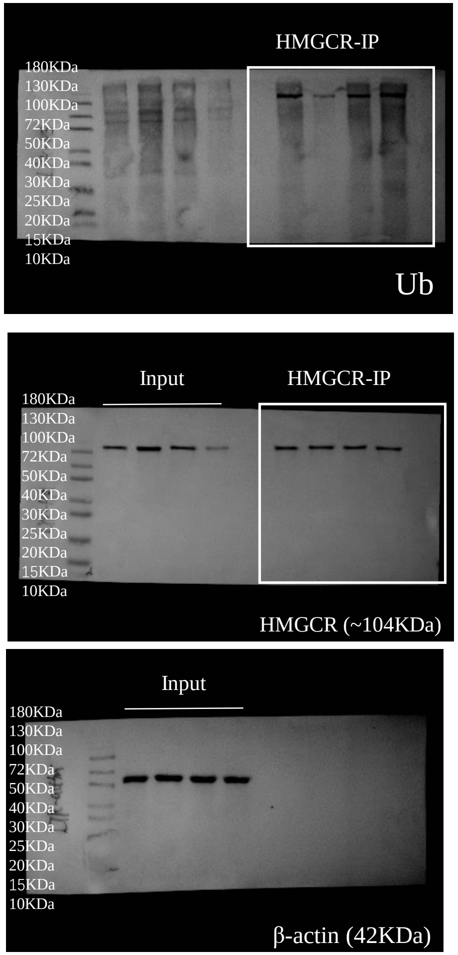

Figure 6A

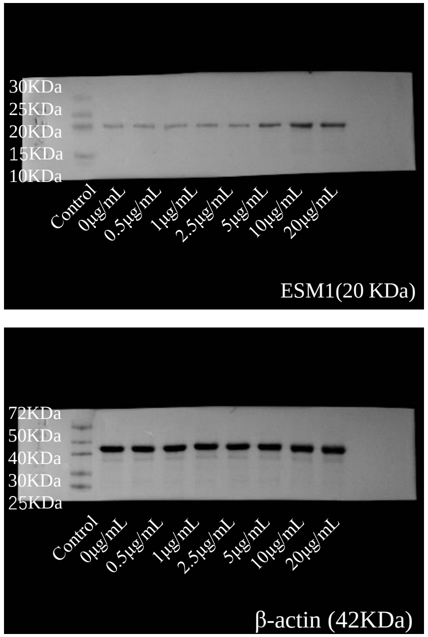

Figure 6B

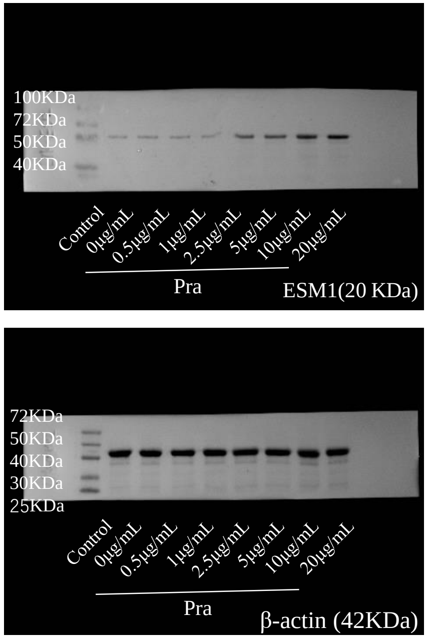

Figure 6C

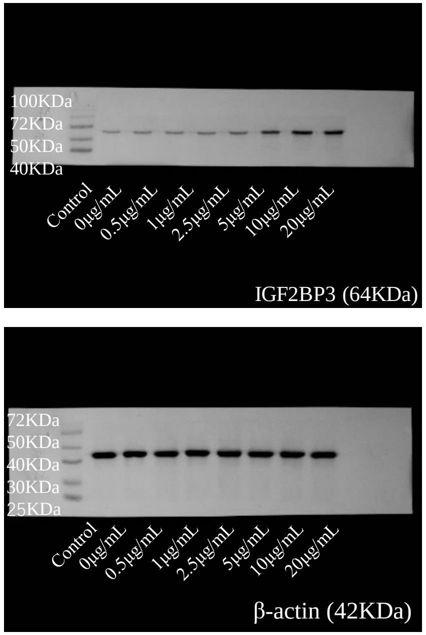

Figure 6D

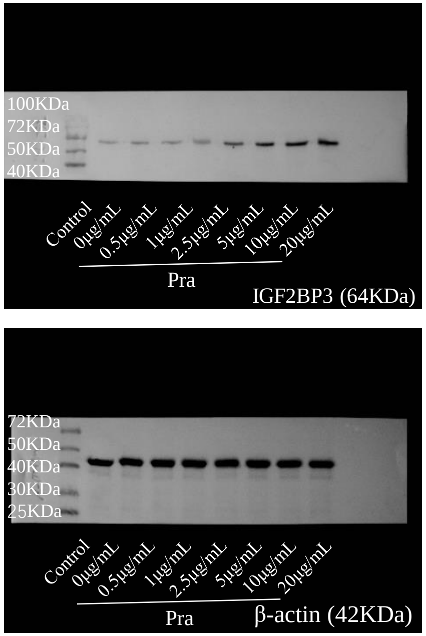

Figure 6K

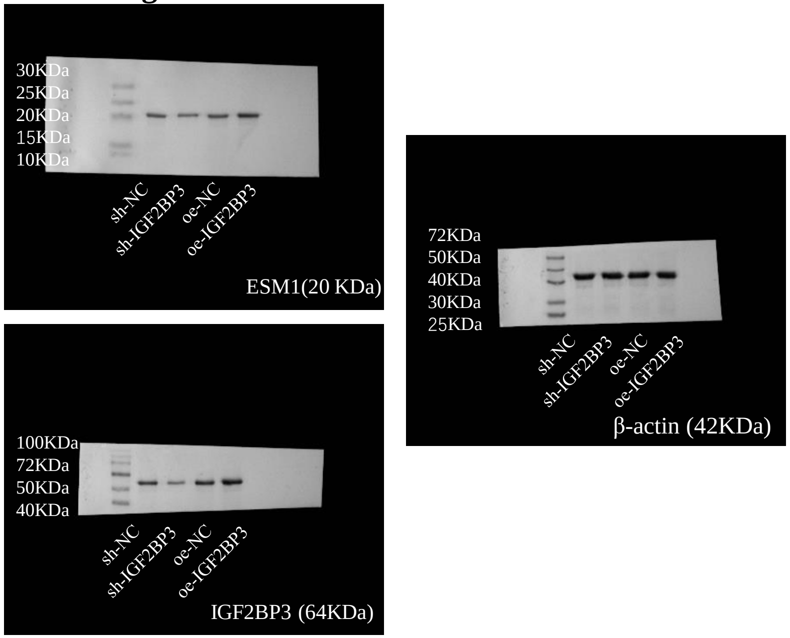

Figure 7C

oe-ESM1 **sh-ESM1**

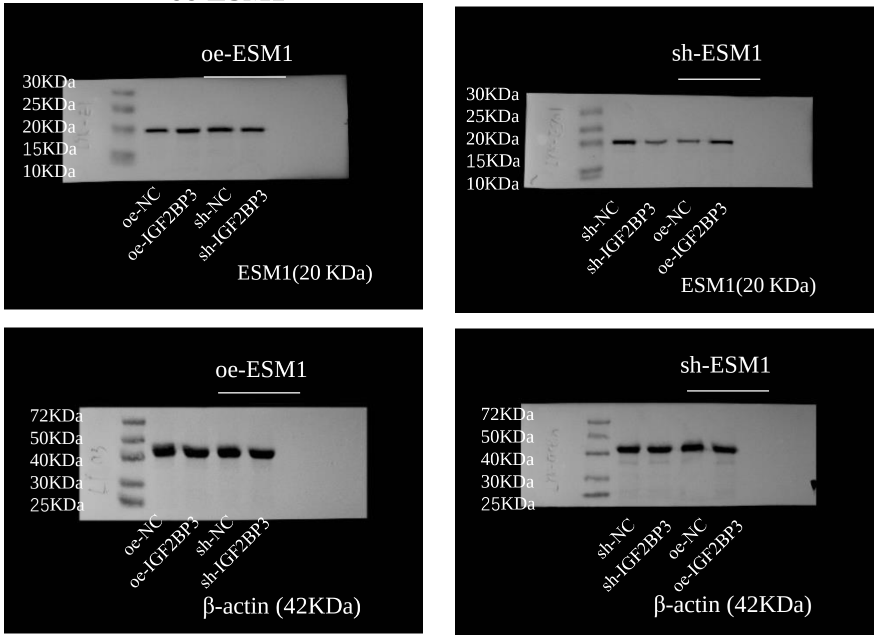

Figure 7D

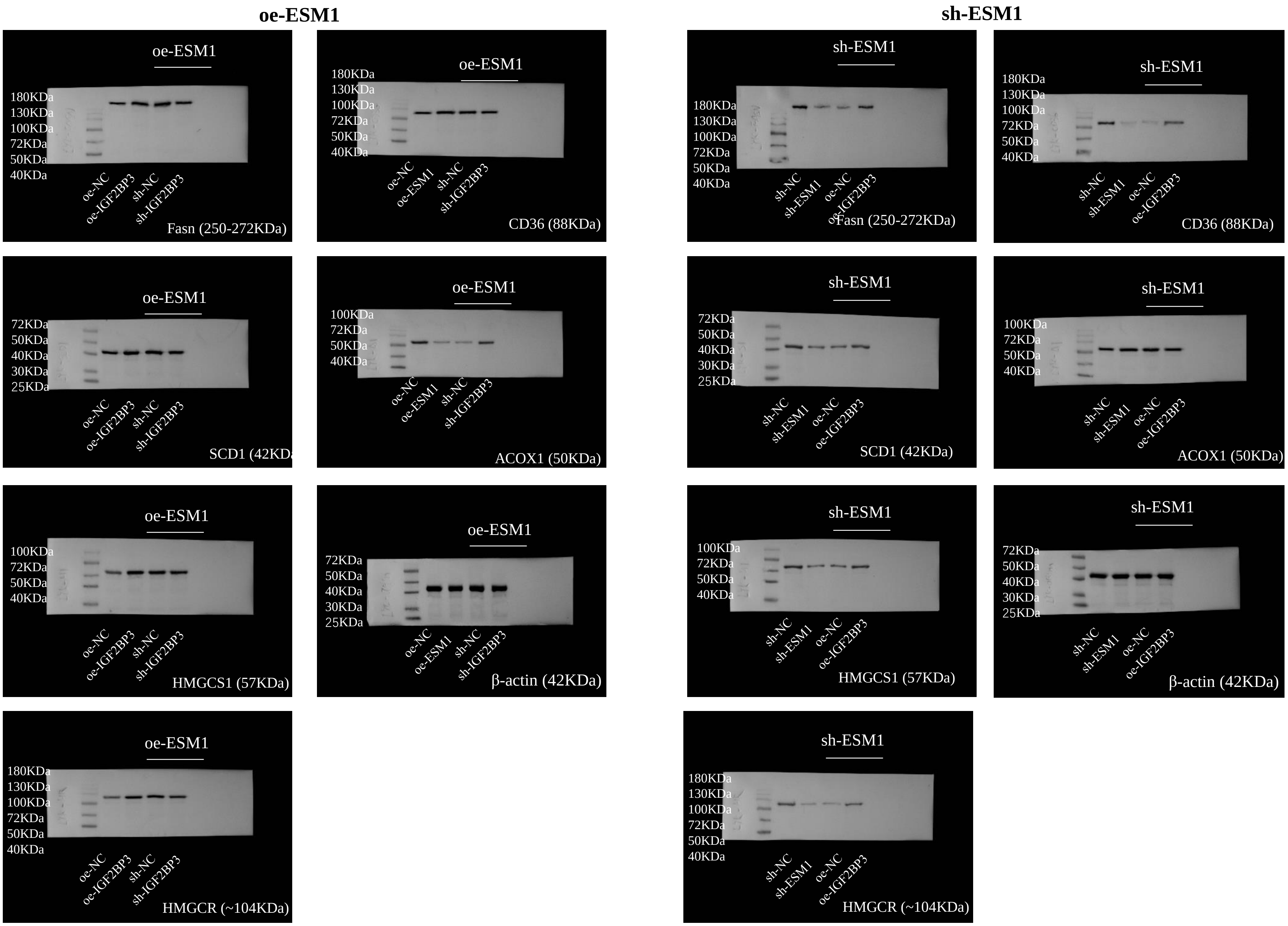
oe-ESM1
sh-ESM1
oe-ESM1
180KDa
130KDa
100KDa
72KDa
50KDa
40KDa
oe-NC
oe-IGF2BP3
sh-NC
sh-IGF2BP3
Fasn (250-272KDa)
oe-ESM1
oe-NC
oe-ESM1
sh-NC
sh-IGF2BP3
CD36 (88KDa)
sh-ESM1
sh-NC
sh-ESM1
oe-NC
oe-IGF2BP3
Fasn (250-272KDa)
sh-ESM1
CD36 (88KDa)
oe-ESM1
72KDa
50KDa
40KDa
30KDa
25KDa
SCD1 (42KD
oe-ESM1
ACOX1 (50KDa)
sh-ESM1
SCD1 (42KDa)
sh-ESM1
ACOX1 (50KDa)
oe-ESM1
HMGCS1 (57KDa)
oe-ESM1
β-actin (42KDa)
sh-ESM1
HMGCS1 (57KDa)
sh-ESM1
β-actin (42KDa)
oe-ESM1
HMGCR (~104KDa)
sh-ESM1
HMGCR (~104KDa)

Figure S3B

A2780

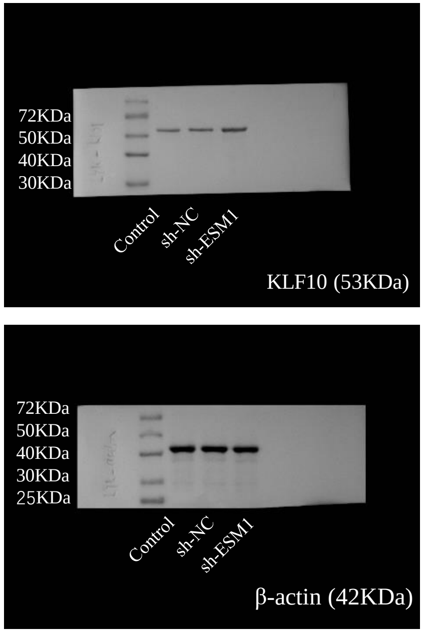

SKOV3

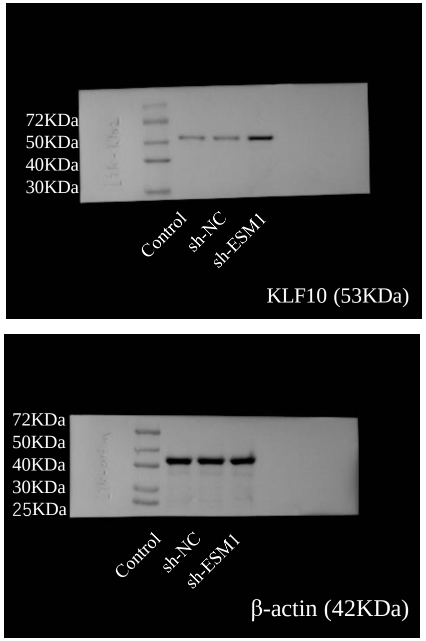

Figure S3C

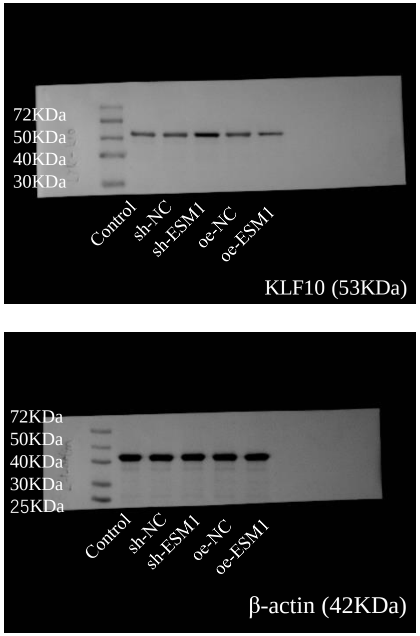

Figure S5A

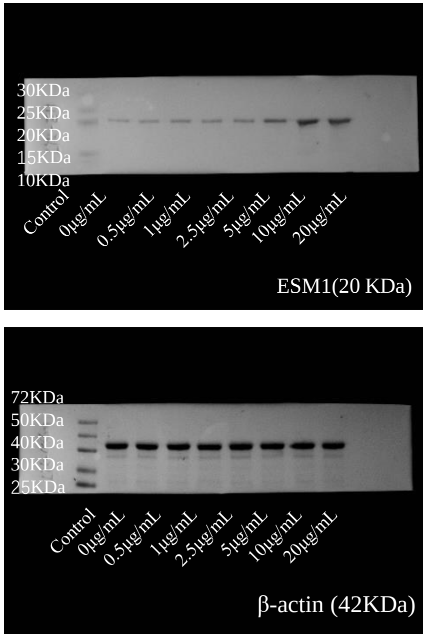

Figure S5B

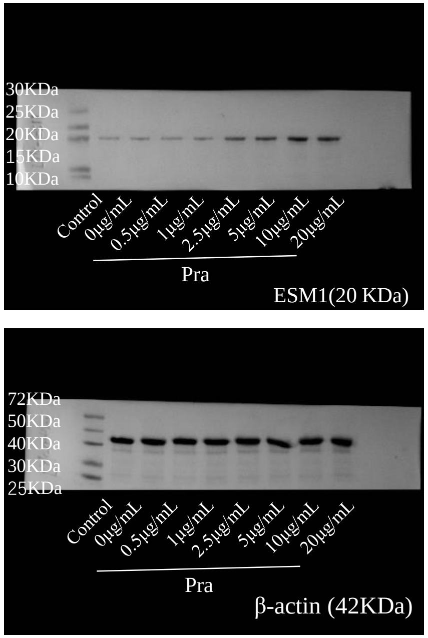

Figure S5C

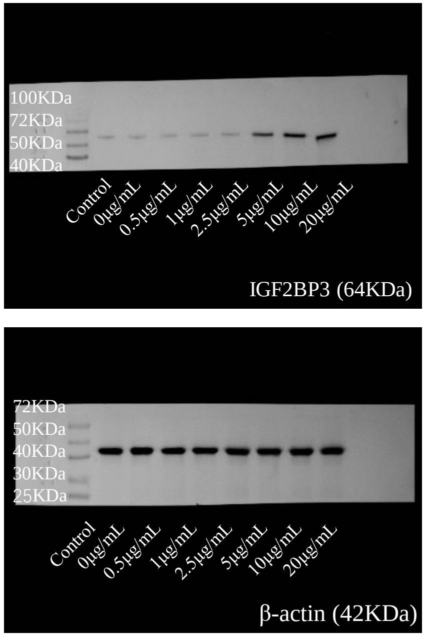

Figure S5D

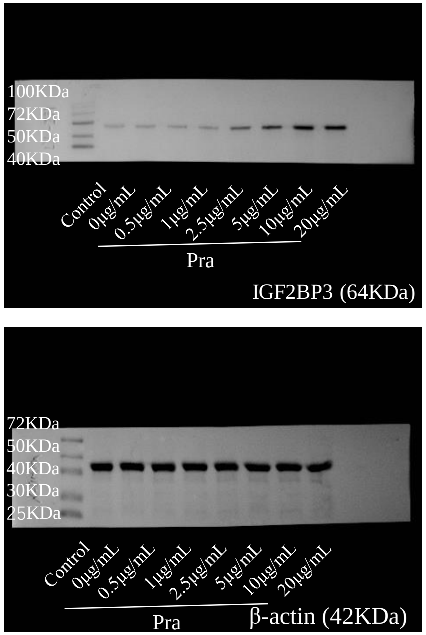

Figure S5K

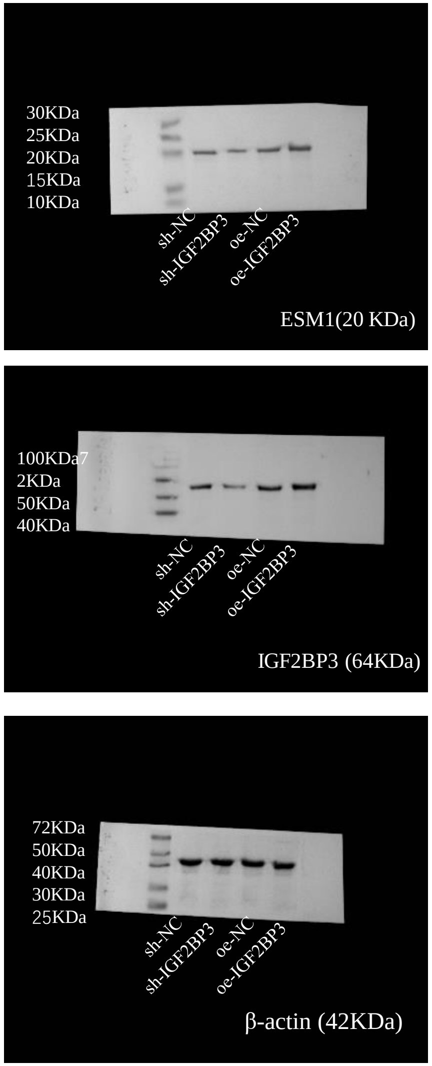

30KDa
25KDa
20KDa
15KDa
10KDa
sh-NC
sh-IGF2BP3
oe-NC
oe-IGF2BP3
ESM1(20 KDa)
100KDa7
2KDa
50KDa
40KDa
sh-NC
sh-IGF2BP3
oe-NC
oe-IGF2BP3
IGF2BP3 (64KDa)
72KDa
50KDa
40KDa
30KDa
25KDa
sh-NC
sh-IGF2BP3
oe-NC
oe-IGF2BP3
β-actin (42KDa)
