# Supplementary material for: IGF2BP3/ESM1/KLF10/BECN1 positive feedback loop: a novel therapeutic target in ovarian cancer via lipid metabolism reprogramming
Source: Cell Death Dis. 2025 Apr 17;16(1):308. doi: 10.1038/s41419-025-07571-7 (PMC12003649; doi:10.1038/s41419-025-07571-7)
Supplement: Supplementary file 2 — Supplementary Information [file 41419_2025_7571_MOESM2_ESM.docx]

**Supplementary Information**

**Supplementary Materials and methods**

**Dual luciferase reporter plasmid information**

The BECN1 gene dual luciferase reporter plasmid was purchased from HonorGene. The plasmid was based on the *BECN1* promoter region and targeted the predicted KLF10 binding site with the following sequence:

GCAGAATATAGTTATGTGCAAGCACTTTGGAAAATGATTCCCAGTGTTAAGGAAGAGACATTAAATAGCTGACACACTCTTAATTCTGTAGTCCCAGTTATGAGTCTCTATCATAAGTAGCCAGCTCTTCATTGCAGGATTATTGTAATCACCCACAGGGGAAATAGTAGAATTTCCAGCGGTAAAAAAATACACTAAGGCAGTACATTTAGTGTAGTGTAATGTAGCCATGATAACTACAATAACTGTGTAGCAACATAGAAAAATGTTAAATTTAAAAAGCAGAAGCCTGGGCAACAAAGTGAGACCCCATCTCTTTTTTTTTTTGAGATGGCGTCTCGCTCTGTCACCGAGGCTGGAGTGCAGTGTGAGACCACATCTCTACAAAAAATTTTAAAAATTAGCTGGGCATGGTAGTGATCACCTGTGGTCCCTGCTACACTGGAGGTTGAAGCAAGAGGATTGCTTGAGCCAGGAAGTCAAATCTGCAGTGAGCCATGTTTGTTTGTTCCGCTTCACTCCAGCCTGGGTAACAGAGTAAGACACTGTCTCAAAATAAAAATAAAATAGACAATACTACATACAATTTTGGGTTAAGCAGTGGTTTCTTTTACACCAAAAGCATAAACATTGGACTTTATTGAAATGAAAAACTTTTGGCCAGGCACATTGGCTCACACCTGTAATCTCAGCACTTTGGGAGGCCACAGTGGGGGATTGCAAGGGGAGATGGGAAATGTTCTAAAACTGGATTATGGTGATAGTTGGGCAACTGTGTAAATTTACTAAAAATTATTGAACTGTACATTTAAAAAGTGTGAGTCTTATGGTATGTAAATTATACCCCATAAAGTTGTTTTTAAAAATGAAGTAAGTCCCTCTGCTCAAGACCCAGTCATCTCATCTCATTCAAAGTGAAAGCCAGAGCTTTACAATCCCTATAAGAGCCTAGGTGGTAGCTCAACACTCTTACCTCCCTCACCCCATTTTCTGTATCTCTTTTCGTTGCCCATCTTCTAGCCACACCAGCCTCTGCTAATCCCCAAACAGGTACCCTCTGTGCTCTTGCTGTTCCCTTGGCCTAGAATGCTCTTCCTTAAGATGCAGGTAAGAATTCCTTCCTCACCTTCTTCAAGCTTTTATTTGAATATCACTTTCTTTTTTTGTTGGTTTTGTGTGTGTGTGTGGGGGGGGGGGGTTTGAGATGGAGTTTCCTTCTGTCGCCCAGGCTGGAGTGCAGTGGCATGATCTCGACTCACTGCAACCTCCGCCTCCGGGGGTCAAGCGATTTTCCTACCCCAGCCTCCTGAGTAGCTGGGATTACAGGCGCACGCCACCATGCCCAGCTAATTGTATTTTTTAGTAGAGACGGGATTTAACCATTTTGGCCAGGCTGGTCTCGAACTCCTGACCTTGTGATCCGCCCGCCTCGGCCTCCCAAAGTGCTGGAATTACAAGCGTGAGCCACCATGCCCGGCCTTTTGTTGTTGCTGTTGTTGTTCTGAGATGGAGCCTTGCCCTGTCGCCCAGGCTGGAGTGCAGTGGCCCGATCTCGGCTCACTGCAACCTCCACCTCCCAGGTTCAAGCGATTCTCCTGCCTCAGCCTCCCGAGTAGCTGGGATTAAGCTGGGATTATAGGCGTGCCCCACCACGCCCGGCTAGTTTTTGTATTTTTAGTAGAGACGGGGTTTCACTGTGTTGGCCAGGCTGGTCTCGAACTCCTGACCTCACGTGATCCGCCCTCCTCGGCCTCCCCAAGTGCTGAGATTACAGGCGTGAGCCACCGCGCCCGCCGCCCCCTGAATTTAGAGAATAGCGGAGCCTCCCCATTCTCTGCGGCCTTGGCTCCTACACTTCCCGTGGTAACCTTGTTCATCCGCTGAAGCCCGCTGCTTTTCCCAGCCCGGCCTCTGGGGGCCGCTGCCGGGCCTGTGAGCCTGTGGACCAGGAGCTCCTGCTGCCGTCGTAGCGTCACGTCCGGTCTCGGGCGGAAGTTTTC

**Mutation site 1:** GGGGGGGGGGGG mutated to CCCCCCCCCCCC

**Mutation site 2:** ATAGGCGTGCCCCACCACGCCCG mutated to TATCCGCACGGGGTGGTGCGGGC

**Supplementary Figures**

**
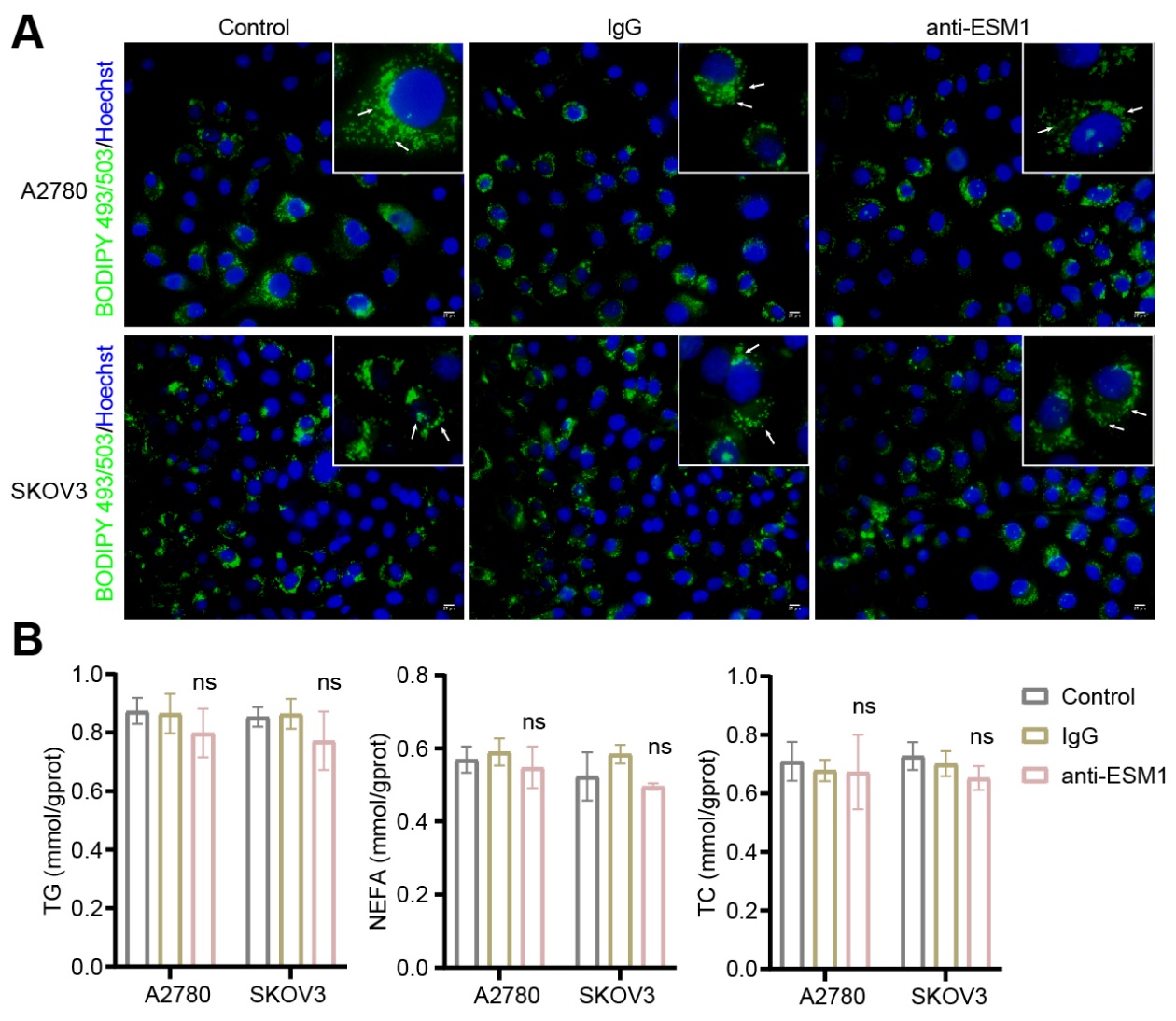
**

**Supplementary Figure 1.** (A) Neutral lipid content was observed by BODIPY 493/503 staining. The white arrows point to the locations where neutral lipid droplets accumulate. (B) The contents of TG, NEFA, and TC were detected by biochemical kits.


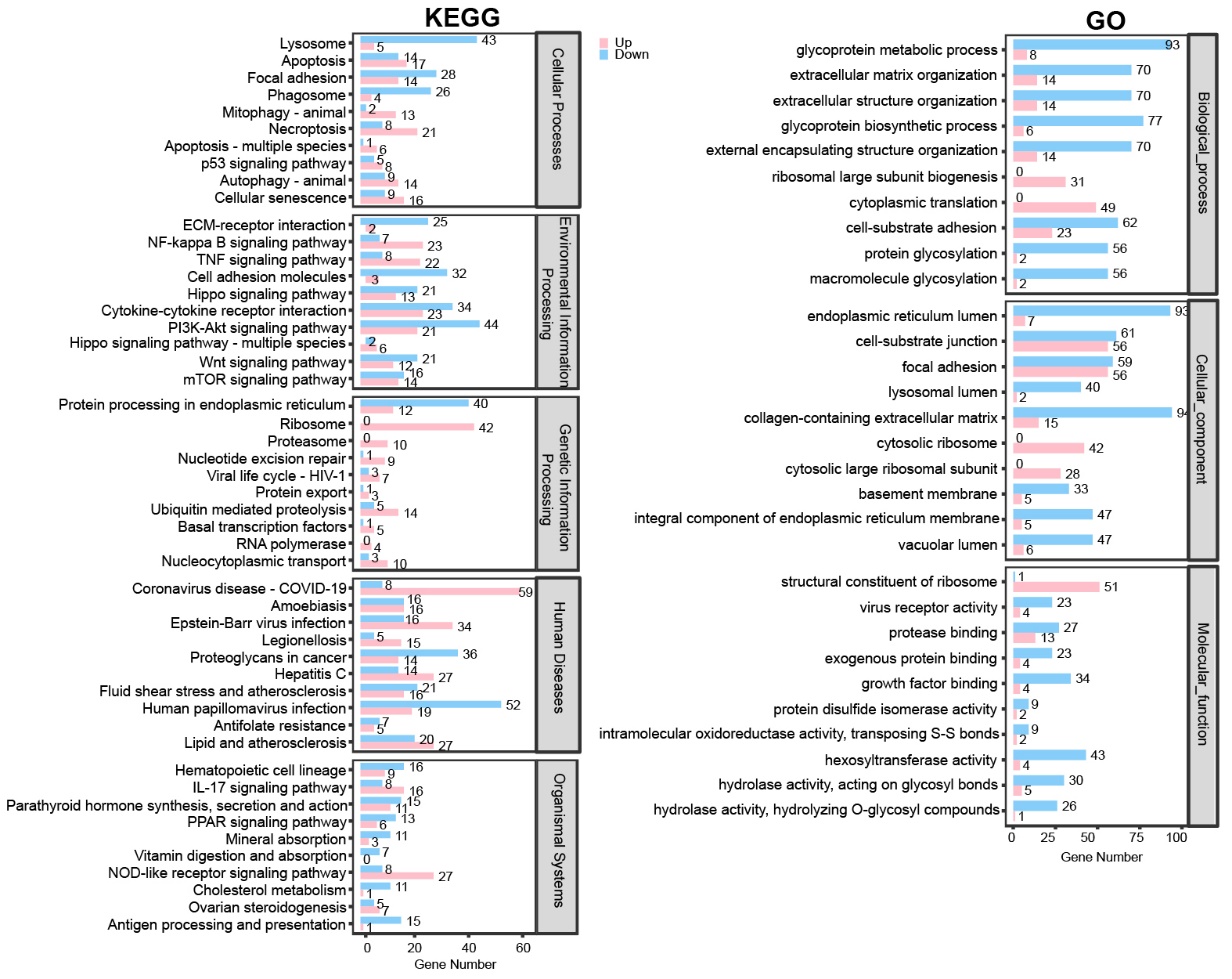


**Supplementary Figure 2.** KEGG and GO functional enrichment analysis of differentially expressed genes with ESM1 silence.


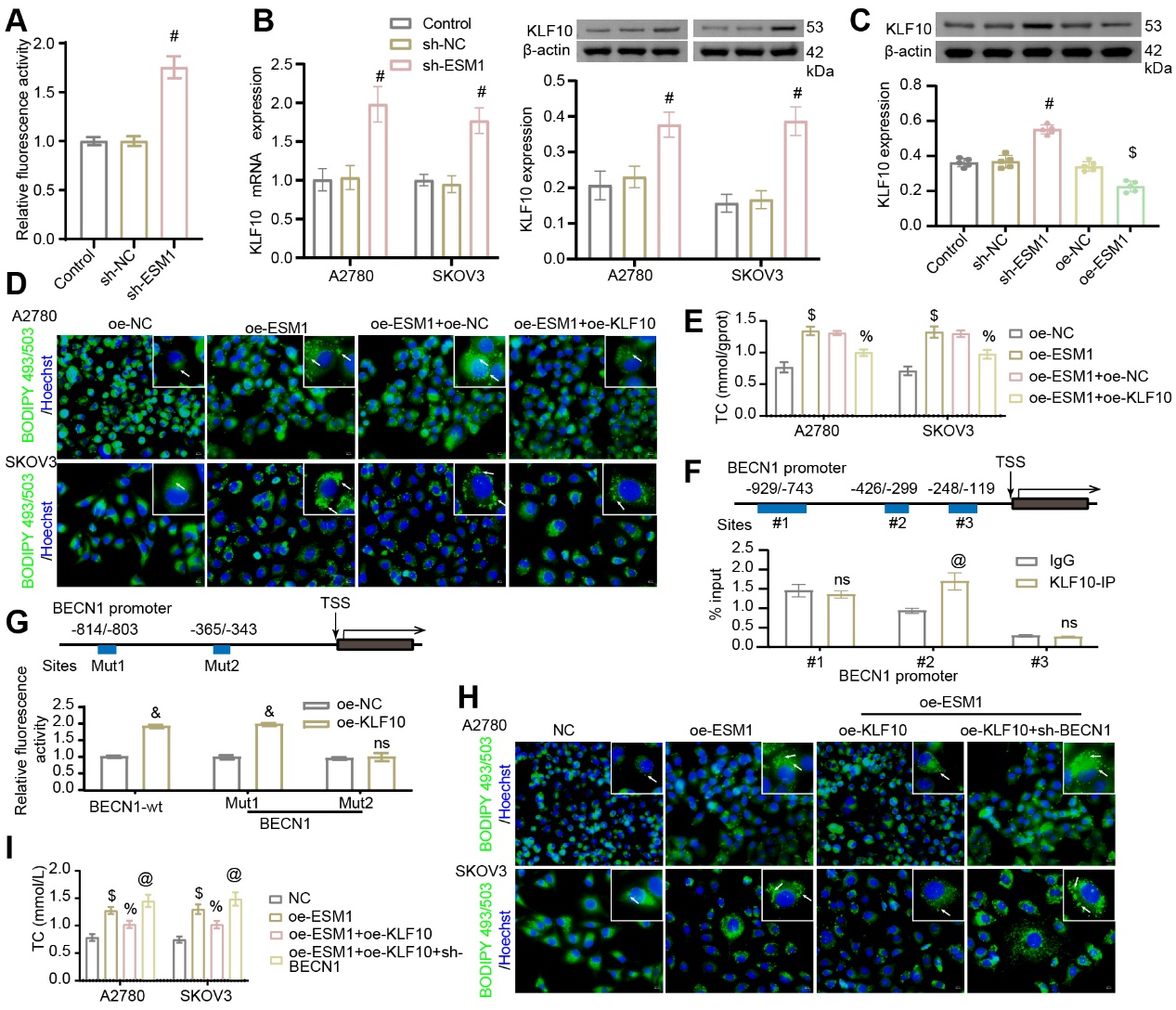


**Supplementary Figure 3. In OC cells, BECN1 regulated by KLF10 is involved in ESM1 regulation of lipid metabolism.** (A) Dual-luciferase reporter assay was applied to verify the promoter activity of BECN1. (B) The level of KLF10 in OC cells was analyzed by RT-qPCR and Western blot. (C) Western blot analysis of KLF10 expression in tumor tissues. # *p*<0.05 vs. sh-NC. $ *p*<0.05 vs. oe-NC. (D) The content of neutral lipids in OC cells was observed by BODIPY 493/503 staining. The white arrows point to the locations where neutral lipid droplets accumulate. (E) Biochemical kits were utilized to analyze the contents of TC in OC cells. $ *p*<0.05 vs. oe-NC. % *p*<0.05 vs. oe-ESM1+oe-NC. (F) ChIP-qPCR to identify the interaction between transcription factor KLF10 and BECN1 promoter. @ *p*<0.05 vs. IgG. (G) Dual-luciferase reporter assay confirmed KLF10 interaction with BECN1. & *p*<0.05 vs. oe-NC. (H) The content of neutral lipids in OC cells was observed by BODIPY 493/503 staining. The white arrows point to the locations where neutral lipid droplets accumulate. (I) Biochemical kits were utilized to analyze the contents of TC in OC cells. $ *p*<0.05 vs. oe-NC. % *p*<0.05 vs. oe-ESM1+oe-NC. @ *p*<0.05 vs. oe-ESM1+oe-KLF10.


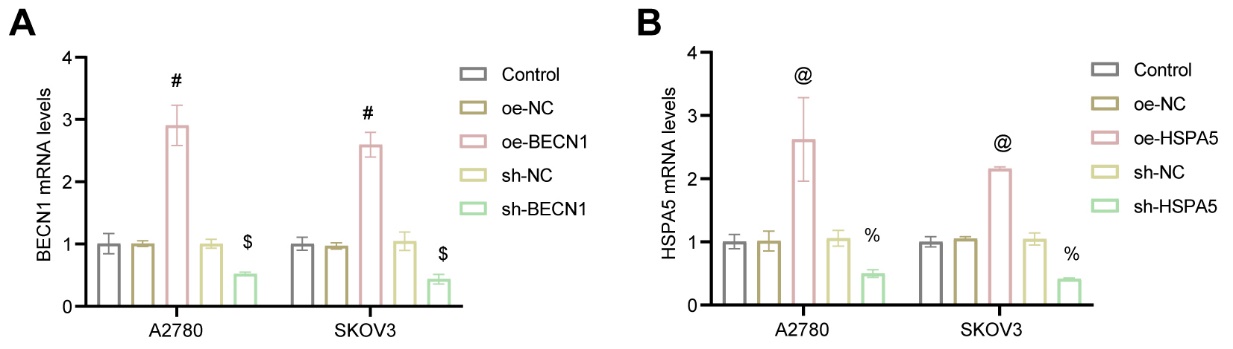


**Supplementary Figure 4.** (A) RT-qPCR and Western blot were applied to verify the overexpression and silencing efficiency of BECN1. # *p*<0.05 vs. oe-NC. $ *p*<0.05 vs. sh-NC. (B) RT-qPCR and Western blot were applied to verify the overexpression and silencing efficiency of HSPA5. @ *p*<0.05 vs. oe-NC. % *p*<0.05 vs. sh-NC.

**
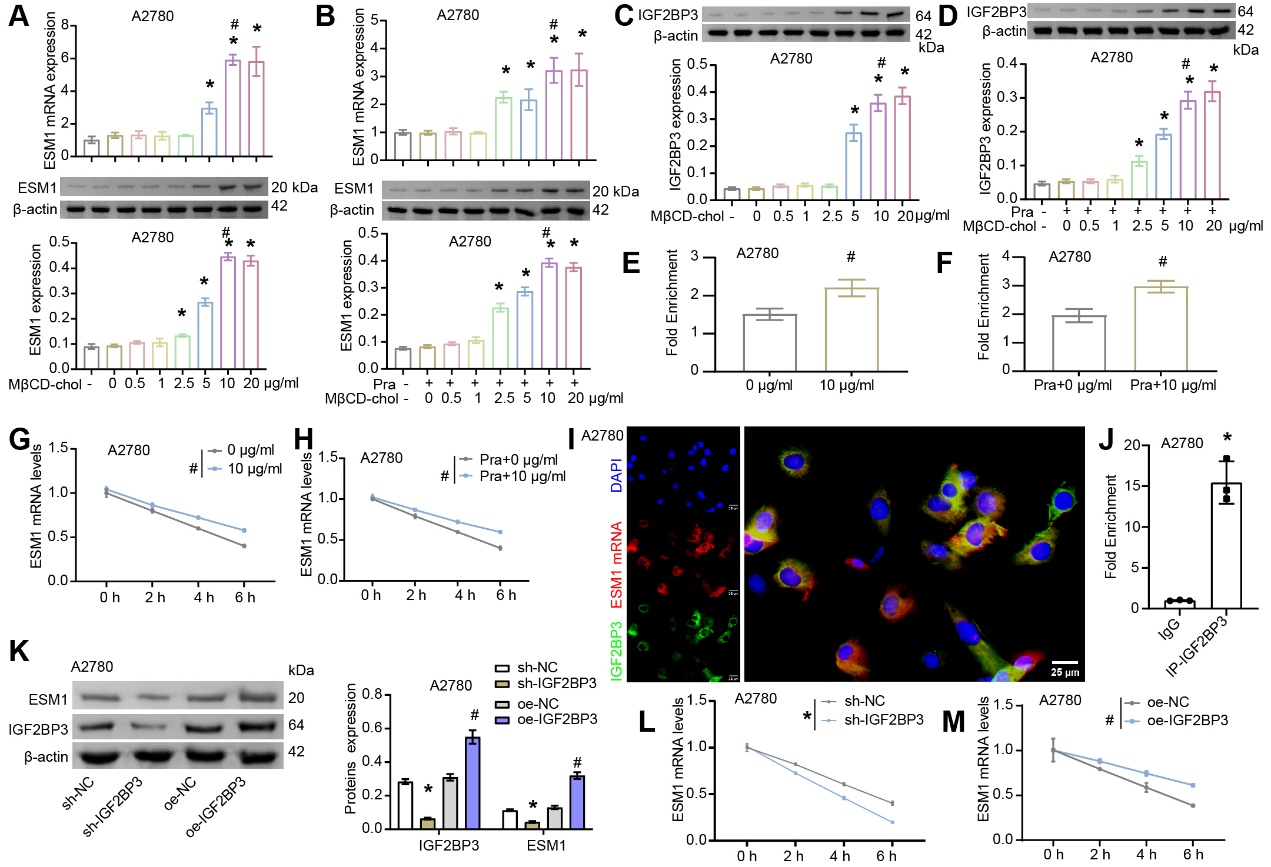
**

**Supplementary Figure 5. The expression of m6A demethylase IGF2BP3 was correlated with cholesterol content in A2780 cells as well as regulated the stability of ESM1 mRNA.** (A-B) RT-qPCR and Western blot analysis of ESM1 level in OC cells. (C-D) Western blot analysis of IGF2BP3 expression in OC cells. * *p*<0.05 vs. 0 μg/mL MβCD-chol. # *p*<0.05 vs. 5 μg/mL MβCD-chol. (E-F) The m6A levels of ESM1 mRNA were evaluated using RIP-PCR. (G-H) The stability of ESM1 mRNA in OC cells was analyzed by actinomycin D treatment assay. # *p*<0.05 vs. 0 μg mL MβCD-chol or Pra+0 μg mL MβCD-chol. (I) The expressions of ESM1 mRNA and IGF2BP3 in OC cells were collocated by FISH and IF analysis. (J) The interaction between ESM1 mRNA and IGF2BP3 was verified by RIP-qPCR. * *p*<0.05 vs. IgG. (K) Western blot analysis of IGF2BP3 and ESM1 levels in OC cells. (L-M) The stability of ESM1 mRNA in OC cells was analyzed by actinomycin D treatment assay. * *p*<0.05 vs. sh-NC, # *p*<0.05 vs. oe-NC.


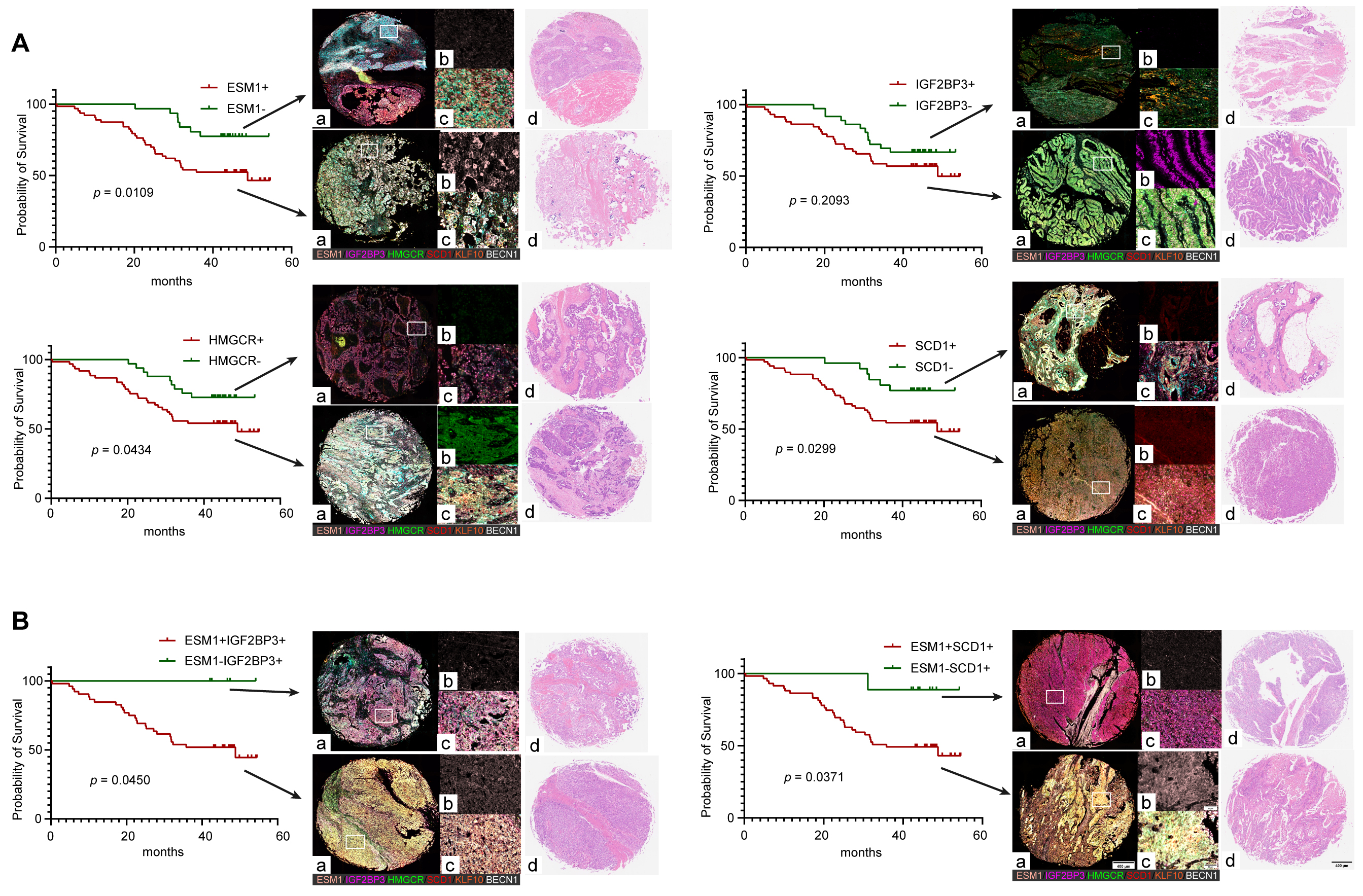


**Supplementary Figure 6. In OC patients, ESM1 regulated IGF2BP3/ESM1/KLF10/BECN1 axis and had a prognostic significance.** (A-B) Comparison of survival curves was conducted by Log-rank (Mantel-Cox) test. The expression levels of ESM1, IGF2BP3, KLF10, BECN1, HMGCR, and SCD1 in tumor tissues of OC patients were measured utilizing microarray analysis. a, merged fluorescence image of panoramic scan (40 ×); b, single indicator expression (400 ×); c, partially enlarged image of a (400 ×); d, HE staining image of panoramic scan (40 ×).
